# OptiPharm: An evolutionary algorithm to compare shape similarity

**DOI:** 10.1038/s41598-018-37908-6

**Published:** 2019-02-04

**Authors:** S. Puertas-Martín, J. L. Redondo, P. M. Ortigosa, H. Pérez-Sánchez

**Affiliations:** 1 Supercomputing - Algorithms Research Group (SAL), University of Almería, Agrifood Campus of International Excellence, ceiA3, Almería, 04120 Spain; 2 Bioinformatics and High Performance Computing Research Group (BIO-HPC), Universidad Católica de Murcia (UCAM), Murcia, 30107 Spain; 3 Centre for Logistics and Heuristic Optimization (CLHO), Kent Business School, University of Kent, Canterbury, CT2 7NZ United Kingdom

## Abstract

Virtual Screening (VS) methods can drastically accelerate global drug discovery processes. Among the most widely used VS approaches, Shape Similarity Methods compare in detail the global shape of a query molecule against a large database of potential drug compounds. Even so, the databases are so enormously large that, in order to save time, the current VS methods are not exhaustive, but they are mainly local optimizers that can easily be entrapped in local optima. It means that they discard promising compounds or yield erroneous signals. In this work, we propose the use of efficient global optimization techniques, as a way to increase the quality of the provided solutions. In particular, we introduce OptiPharm, which is a parameterizable metaheuristic that improves prediction accuracy and offers greater computational performance than WEGA, a Gaussian-based shape similarity method. OptiPharm includes mechanisms to balance between exploration and exploitation to quickly identify regions in the search space with high-quality solutions and avoid wasting time in non-promising areas. OptiPharm is available upon request via email.

## Introduction

The discovery of new drugs is a very expensive process, frequently taking around 15 years with success rates that are usually very low^1,2^. Many experimental approaches have been used for discovering new compounds with the desired pharmacological properties, ranging from traditional medicine^3,4^ to High Throughput Screening (HTS) infrastructures^5,6^. The latter is mostly used by the Pharma Industry, but little by academic research groups; in other words, its application is not widespread outside the industrial domain. In order to avoid these limitations, new techniques based on principles of Physics and Chemistry were developed about three or four decades ago for the computer simulation (mainly using high-performance computing architectures) of systems of biological relevance^7,8^. Computational chemistry was later applied for processing large compound databases, and also for predicting their bioactivity or other relevant pharmacologic properties. Using this approach, it was shown that it was possible to use such computational methodology to pre-filter compound databases into much smaller subsets of compounds that could be characterized experimentally. This idea was named Virtual Screening (VS), and it reduces the time needed and expenses involved when working on drug discovery campaigns^9,10^. Nonetheless, the accuracy of the predictions made with VS methods still needs to be improved to avoid discarding promising compounds or providing erroneous signals and the time needed for their calculations still needs to be reduced. The inaccuracies in the predictions of VS methods are mostly due to the simplifications used in their scoring functions^11^.

VS methods can be divided into Structure-Based (SBVS) and Ligand-Based (LBVS) methods. When the structure of the protein target is known, SBVS can be applied, and methods such as molecular docking^12^ and Molecular Dynamics^13^ are employed. But the number of already resolved crystallographic structures is still insufficient^14^, so SBVS methods cannot always be applied. Another option is to use LBVS methods, where only data about known compounds with desired properties are used to derive new improved ones. In practice, whether SBVS or LBVS methods should be used, or even both at the same time, will depend on the specific drug discovery project.

This study focuses on LBVS methods, which can be divided into several categories^15^ such as pharmacophore methods^16,17^, shape similarity methods (SSM)^18^, QSAR^19^, Machine Learning^20^, atom-based clique-matching such as SQ/SQW^21^ and Lisica^22^, property-based (USR^23^) or atom distribution triplet based (Phase-Shape^24^).

In SSM, a large database of compounds is processed against a molecular query, to provide information concerning which of the molecules from the database is geometrically similar, in terms of global molecular shape, to the input molecule used. Indeed, different strategies exist for shape calculation. One of the most widely used is the Gaussian^25^ model. Tools such as ROCS^26^, WEGA^27^, SHAFTS^28^ and Shape-IT^29^ use it.

The main differences between SSM reside in the accuracy of the predictions. It has been demonstrated that, depending on the compound dataset, some methods perform better than others^30^, but there is currently no one-size-fits-all approach that can be considered first choice for any molecular dataset. Besides, the computational time needed for the calculations is also of the utmost importance.

Among the previously commented SSM methods, we consider WEGA to be the state of the art in terms of accuracy of the predictions, while ROCS is considered to be the state of the art in terms of computational speed. For achieving such performance, ROCS introduced a number of drastic short-cuts for efficiency for computing overlap volumes between molecules^31^. For instance, all hydrogen atoms are ignored as they make very small contribution for the overall molecular shape, and all heavy atoms are set with equal radii. Besides, the most critical simplification in ROCS is that the shape density function of each molecule contains only the first-order terms, and all higher order terms in the original Gaussian approach^25^ are omitted. This significantly simplified ROCS computations but also received criticism for the inaccuracy of this approximation^23^; mainly that the molecular volumes are significantly overestimated. And since the Gaussian shape algorithms are widely used in various VS methods, it is important to avoid errors introduced to the shape similarity calculation due to this overestimation of the volumes.

WEGA was the first method that partially solved some of these ROCS issues by avoiding the use of only first-order terms and incorporating more terms, at the expenses of increasing computation costs, but increasing accuracy of the calculations, which is desirable in the drug discovery context.

In this work, we introduce a novel SSM method named OptiPharm, which introduces a new optimization scheme that can be adapted through extensive parameterization to relevant features of molecular datasets, such as average size, shape, etc. In other words, OptiPharm is an evolutionary method for global optimization, which can be parametrized to different aims. SSM methods with extensive parameterization at the search level have not been practically explored in the VS context. The most of techniques are local optimizers which do not sufficiently explore the search space. As the results we later show, making an effort to deeply explore the whole search space can be of a great interest to increase hit rates in drug discovery projects.

## Method

This section describes the main idea behind shape similarity calculations and its application in the drug discovery process using the new OptiPharm method. Next, the optimization algorithm used in similarity screening calculations is presented and, finally, the benchmarks used in this study are explained in detail.

### Shape Similarity

The similarity score between molecules A and B is computed as the overlapping volume of their atoms. In particular, to compare the results obtained by OptiPharm with those achieved by WEGA, the similarity function is implemented as in WEGA^27^. For the sake of completeness, this function is written in the following form:

1 $${V}_{AB}^{g}=\sum _{i\in A,j\in B}\,{w}_{i}{w}_{j}{v}_{ij}^{g}$$

where *w*_*i*_ and *w*_*j*_ are weights associated with the atoms *i* and *j*, respectively. This weight is calculated by solving the following mathematical expression:

2 $${w}_{i}=\frac{{v}_{i}^{g}}{{v}_{i}^{g}+k\,{\sum }_{j\ne i}\,{v}_{ij}^{g}}$$

where *k* is a universal constant, which is set to 0.8665, *v*_*i*_ is the volume of atom *i*, whose value is computed as ${v}_{i}=\frac{4\pi {\sigma }^{3}}{3}$, similarly to how it was done in the original work of WEGA^27^.

Finally, the overlapping ${v}_{ij}^{g}$ is represented as a product of Gaussian representations:

3 $${v}_{ij}^{g}=\int \,{g}_{i}(r){g}_{j}(r)d{\bf{r}}=\int \,p{e}^{-{(\frac{3p{\pi }^{1/2}}{4{\sigma }_{i}^{3}})}^{2/3}{({\boldsymbol{r}}-{{\boldsymbol{r}}}_{{\boldsymbol{i}}})}^{2}}p{e}^{-{(\frac{3p{\pi }^{1/2}}{4{\sigma }_{j}^{3}})}^{2/3}{({\boldsymbol{r}}-{{\boldsymbol{r}}}_{{\boldsymbol{j}}})}^{2}}d{\bf{r}}$$

where *p* is a parameter that controls the softness of the Gaussian spheres, i.e., the height of the original Gauss function, and *σ* is the radius of the atom. More precisely, the radius represents the well-known van der Waals radius. The values associated to those two parameters are obtained by empirical knowledge. For the problem under consideration, the same figures proposed in WEGA^27^ are considered.

Notice that the score obtained from Equation 1 depends on the number of atoms of the two compared molecules, i.e., the higher this number, the longer the value of ${V}_{AB}^{g}$. In reality, it lies in the interval [0, +inf). To be able to measure the grade of similarity between compounds, independently of the number of atoms that compose them, the Tanimoto Similarity^32^ value is computed:

4 $$Tc=\frac{{V}_{AB}}{{V}_{AA}+{V}_{BB}-{V}_{AB}}$$

where *V*_*AA*_ and *V*_*BB*_ is the self-overlap volume of molecules A and B, respectively. It has a value in the range $[0,1]$, where 0 means there is no overlapping, and 1 means the shape densities of both molecules are the same.

### Previous approaches

WEGA is a local optimizer conceived to maximize the overlapping between two molecules A and B, given as input parameters. To direct the search, it computes the derivate of the objective function *Tc*, which specifically considers Equation 1. It means that WEGA can be only applied when the similarity of two molecules is measured by means of such an equation.

WEGA starts the search with an initial solution and moves it from neighbor to neighbor as long as possible while increasing the objective function value. The main advantage of WEGA is its ability to find a solution in a sufficiently short period of time. On the contrary, its main drawback is its difficulty to escape from local optima where the search cannot find any further neighbor solution that improves the objective function value, i.e., the quality of the final solution closely depends on the considered starting ligand pose, obtained from the conformation of the molecular query. To deal with this drawback and to increase its probability of success, WEGA considers more than a single starting point. More precisely, it applies the local optimizer from four different poses. The first one is obtained by aligning and centering the two input molecules at the origin of the coordinates. The remaining ones are obtained by rotating the first one 180 grades at each axis^27^.

The interested reader can revise literature^33–35^ for the research progress of WEGA algorithm and some of its applications. In this work, we consider that it is possible to find a better trade-off between quality of the solution and computing time.

### Optimization algorithm

OptiPharm is an evolutionary global optimizer, available upon request. It can be considered a general-purpose algorithm, in the sense that it can be used to solve any optimization problem that involves the computation of the similarity of two compounds given as input parameters. In other words, it is independent of the objective function used to measure the similarity between two given molecules. Nevertheless, in this work, its performance is illustrated by solving a maximization problem which consists on finding the ***s*** solution which maximizes the *Tc* function previously defined.

OptiPharm is a global optimization method in the sense that it makes an effort to analyze the whole search space looking for promising areas where the local and global optima can be. In other words, instead of focusing on a set of pre-specified starting points, as WEGA does, it applies procedures to find promising subareas of the search space, which will be deeper analyzed during the optimization procedure. OptiPharm applies procedures based on species evolution to gradually adjust one of the molecules (the query) to the other one (the target), which remain fixed during the optimization procedure.

A solution ***s*** represents the rotation and translation to be accomplished by the query. More precisely, ***s*** is a quaternion of the form ***s*** = (*θ*, ***c***_**1**_, ***c***_**2**_, **Δ**), where *θ* is the rotation angle to be carried out over a rotation edge defined by the points ***c***_**1**_ = (*x*_1_, *y*_1_, *z*_1_) and ***c***_**2**_ = (*x*_2_, *y*_2_, *z*_2_), and **Δ** = (Δ*x*, Δ*y*, Δ*z*) represents a displacement vector. It should be borne in mind throughout that a quaternion indicates the rotation and the translation applied to the variable molecule from its initial state.

**Figure 1:**
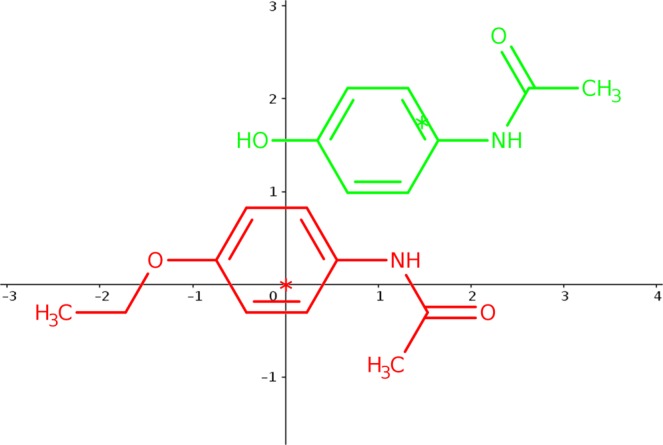
The correct bounding of the parameter **Δ** prevents the evaluation of poor quality solutions, such as that considered in this figure, where no overlapping exists and hence the shape similarity of both molecules is equal to zero.

The parameters associated to a quaternion ***s*** are bounded. Since each pair of input compounds can have different sizes, the corresponding limits are dynamically computed by OptiPharm, for each particular instance. To do so, the 3D boxes containing the input compounds are calculated. Then, the bound values for both ***c***_**1**_ and ***c***_**2**_ are set to the borders of the box containing the variable molecule. Notice that the same axis can be given by an infinite number of two coordinates. In this way, redundancy is prevented, which is very important from an optimization point of view, since exploring the same solutions several times makes the algorithm inefficient. The interval of **Δ** is set to [−***maxD***, ***maxD***], being ***maxD*** the maximum difference between the boxes. This avoids the evaluation of situations where no overlapping exists between molecules, and the similarity between them is clearly zero (see Fig. 1). Finally, the angle *θ* is always set in the interval [0, 2*π*], independently of the compounds considered as input parameters.

The bound values of the quaternion components define a multidimensional search space (or feasible region) with multiple local and global optima.

**Figure 2:**
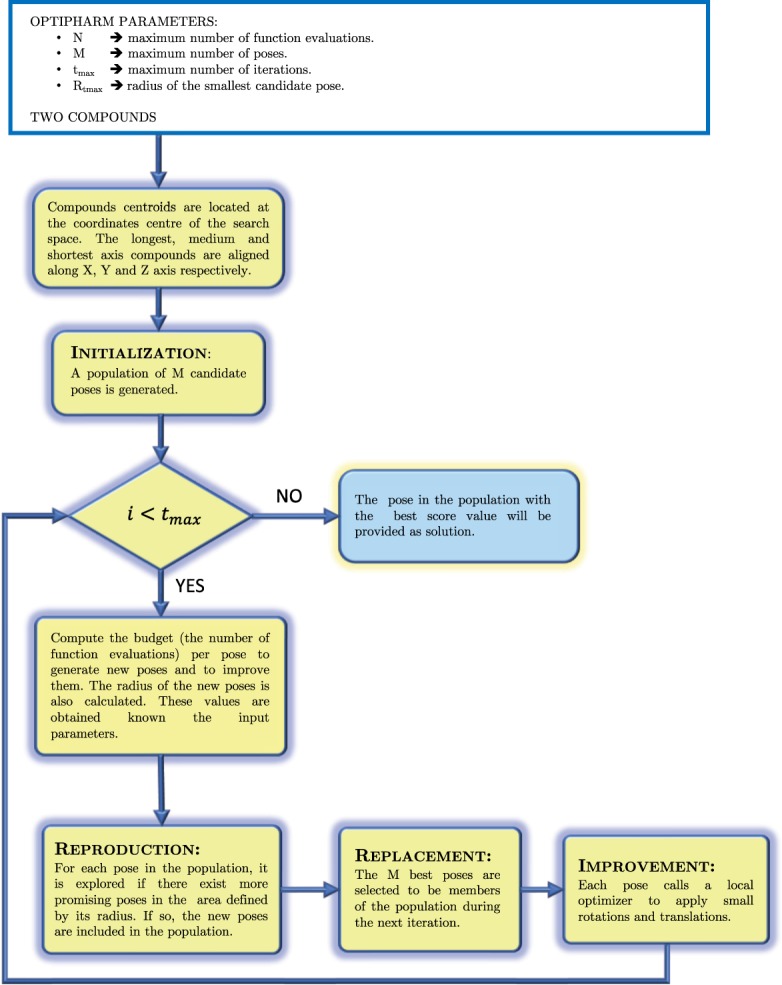
OptiPharm algorithm structure.

OptiPharm is a new metaheuristic for global optimization. OptiPharm includes mechanisms to detect promising subareas of the search space and to discard those in which no global optima are expected to be found. In other words, instead of focusing on some fixed starting solutions, OptiPharm attempts to detect new ones which have the potential to become local or global optima. To do so, OptiPharm initially works on a set of *M* solutions (quaternions), called *population*. The quaternions can be considered as independent starting points on which OptiPharm applies reproduction procedures based on natural evolution. The term *independent* signifies that a point has the ability to discover new promising poses (in this work we use the concept of pose as rigid body rotations and translations obtained from starting conformation of query compound) without the participation of the rest of the population. As a consequence, offsprings of new promising solutions can appear. Then, from among all the existing poses, the best *M* solutions will be promoted to the next stage, where they are improved by means of a local optimizer. This reproduction-replacement-improvement sequence is repeated until a number of iterations *t*_*max*_ is achieved (see Fig. 2).

But the real strength of OptiPharm lies on the concept of radius: each solution in the population has an associated radius value, which determines a multidimensional subarea of the search space. It can be understood as a window, where the reproduction and improvement methods are applied. The radius associated to a pose depends on the iteration *i* where it has been discovered. More precisely, the radius *R*_*i*_ of a new point, found during the reproduction procedure at iteration *i*, comes from an exponential function that decreases as the index level (cycles or generations) increases, and which depends on the initial domain landscape (the radius at the first level, *R*_1_) and the radius of the smallest candidate solution ${R}_{{t}_{max}}$, which is given as input parameter. This radius mechanism, designed as a balance between *exploration* and *exploitation*, is inherited from UEGO, a general optimization method widely used in the literature with promising results^36^.

**Figure 3:**
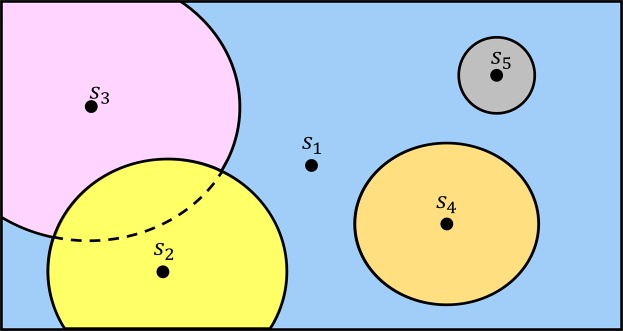
Several solutions with different radii can coexist simultaneously. Therefore, at the same stage of the optimization procedure, new promising regions are systematic analyzed, while others are examined thoroughly. This figure illustrates an example for a 2-dimensional case.

During the execution of OptiPharm, several candidate solutions with different radii can coexist simultaneously which means that the method is able to analyze both big and small subregions at the same stage of the optimization procedure as it looks for valuable new solutions (see Fig. 3).

Apart from the maximum number of starting solutions *M*, the number of iterations *t*_*max*_ and the smallest radius value ${R}_{{t}_{max}}$, OptiPharm has another input given parameter: the maximum number of function evaluations for the whole optimization procedure, *N*. These function evaluations are distributed among the candidate solutions at each iteration, in such a way that each one has a budget to generate new solutions and to improve them. These budgets are mathematically computed by means of equations that depend on the previously mentioned input parameters. Again, this idea has been borrowed from UEGO^36^.

In a previous work^37^ the effects of the different parameters of UEGO and, hence, of OptiPharm were analyzed. Moreover, some guidelines to fine-tune the parameters depending on the problem to be solved were also proposed.

Finally, it should be noted that, unlike most heuristics in the literature, the termination criteria of OptiPharm is not based on the number of function evaluations *N*, but on the number of iterations *t*_*max*_. This point is important since the number of function evaluations consumed by OptiPharm depends on the particular case being solved. In other words, OptiPharm adapts itself to the complexity of the problem considered.

In the following subsubsections, the key stages of OptiPharm are explained.

#### Initialization method

**Figure 4:**
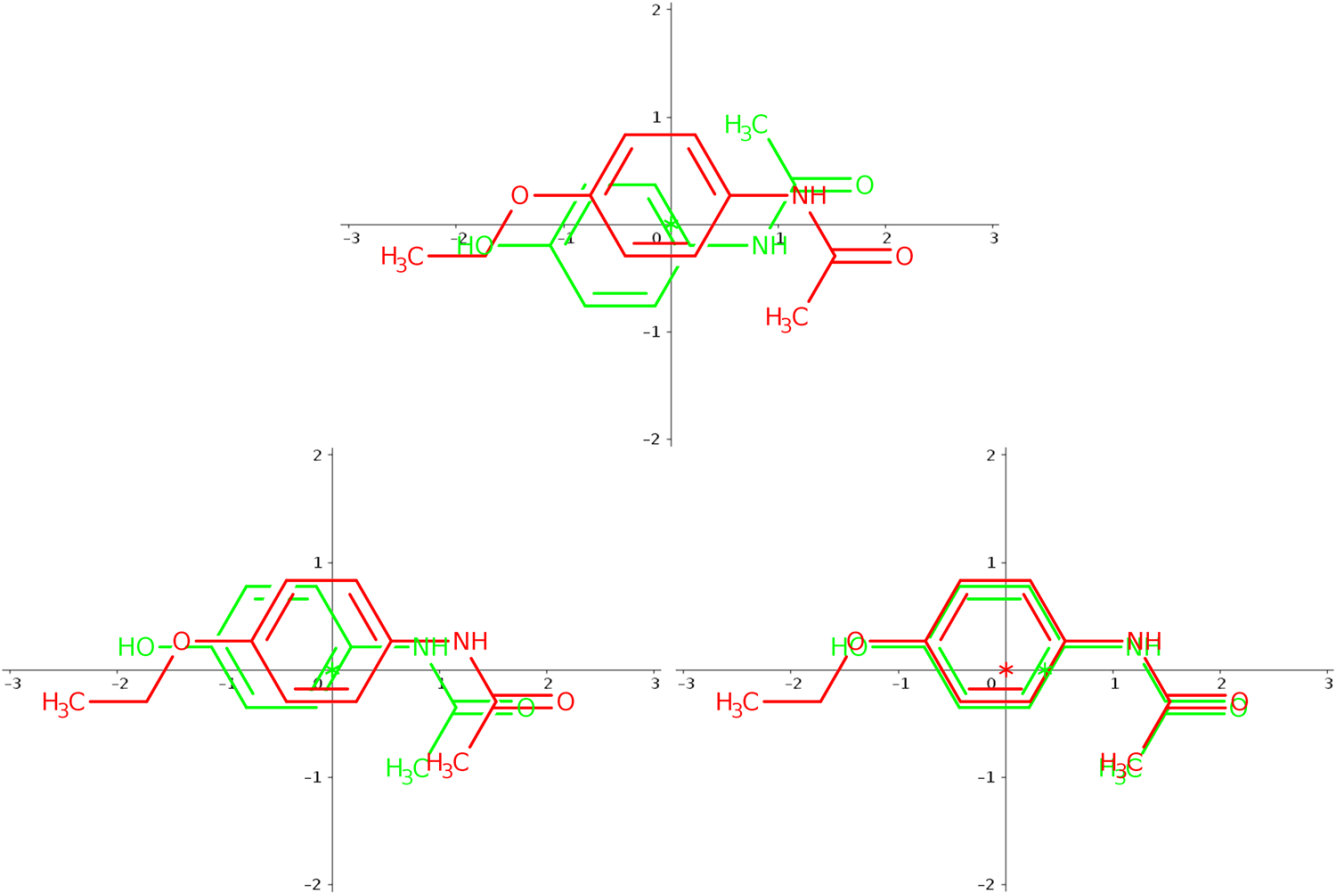
Initially both molecules are aligned and centered at the origin of the coordinates (see figure above). The variable molecule is depicted in green, while the target is represented in red. Then, OptiPharm applies procedures based on species evolution to gradually adjust the variable molecule to the target. The two figures below show intermediate solutions obtained by OptiPharm when, from the initial state (top), a rotation is carried out (left) and a consecutive translation is accomplished (right).

In the initialization phase, the two input molecules are aligned and centered at the origin of the coordinates (see Fig. 4). Then, from this initial situation, a population of *M* poses is composed. The first pose represents this initial stage, i.e. the former candidate solution will be equal to ***s***_**1**_ = (*θ*, ***c***_**1**_, ***c***_**2**_, **Δ**) = (0, (0, 0, 0), (0, 0, 0), (0, 0, 0)), indicating than the molecule to be optimized is not moved with respect to the target, which remains fixed. Three more initial poses are obtained by rotating the variable molecule *π* radians at each axis (always from the initial state), resulting in the following candidate solutions ***s***_**2**_ = (*π*, (1, 0, 0), (0, 0, 0), (0, 0, 0)), ***s***_**3**_ = (*π*, (0, 1, 0), (0, 0, 0), (0, 0, 0)) and ***s***_**4**_ = (*π*, (0, 0, 1), (0, 0, 0), (0, 0, 0)). Finally, in order to introduce some randomness and prevent a possible drift to local optima, *M* − 4 molecular poses, with all their randomly obtained parameters, are also included.

**Figure 5:**
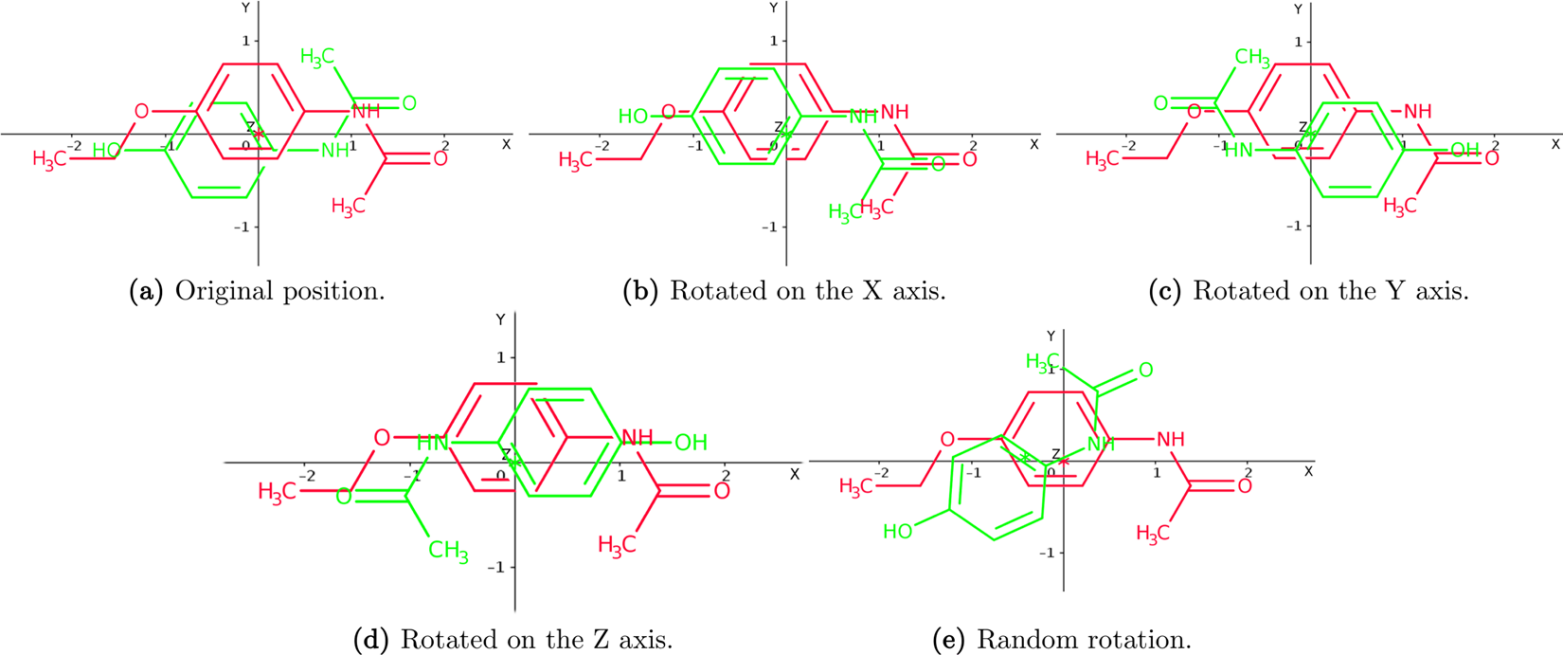
Initial solutions for a case with *M* = 5: (**a**) ***s***_**1**_, initial situation; (**b**) ***s***_**2**_, obtained when rotating ***s***_**1**_ *π* rad at *x*-axis; (**c**) ***s***_**3**_, obtained when rotating ***s***_**1**_ *π* rad at *y*-axis; (**d**) ***s***_**4**_, obtained when rotating ***s***_**1**_ *π* rad at *z*-axis; (**e**) ***s***_**5**_, all the parameter (*θ*, ***c***_**1**_, ***c***_**2**_, **Δ**) are randomly computed in the limits dynamically calculated by OptiPharm, for this particular instance.

Figure 5 shows the five initial solutions achieved for a particular instance with *M* = 5. As can be seen, there is always some overlap between both molecules. Consequently, the objective function is always greater than zero, while the radius value associated to all the initial poses is equal to *R*_1_. Notice that such a value is equal to the diameter of the search space.

#### Reproduction method

**Figure 6:**
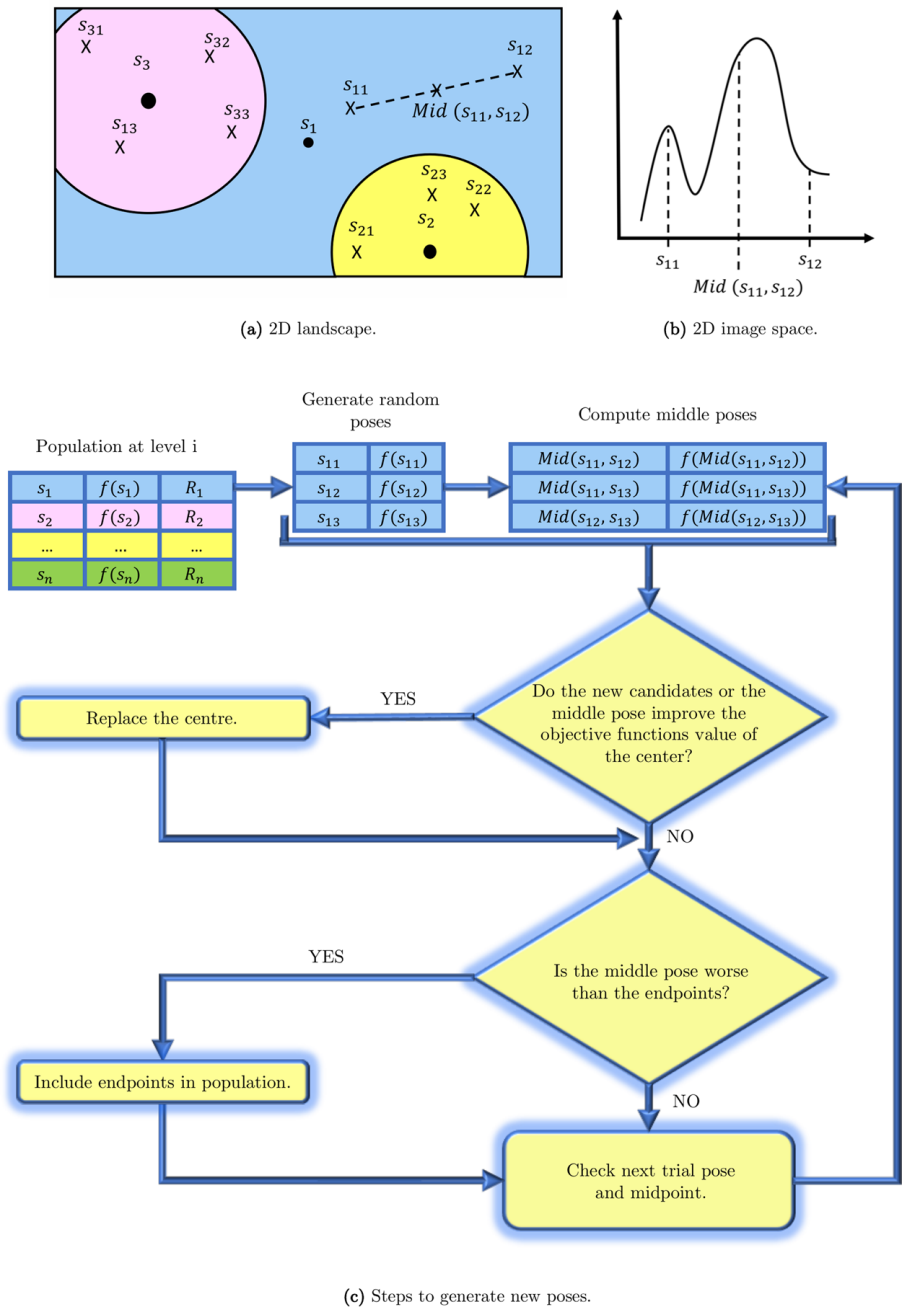
Reproduction method.

The reproduction method is in charge of exploring the different subareas defined by the radius of each pose ***s*** in the population (see Fig. 3). The idea is to find new promising solutions which can evolve toward local or global optima at later phases of the algorithm. Each subarea is analyzed independently of the remaining ones. The process is as follows:

From each pose ***s***_***i***_ in the population, new candidate solutions ***s***_***ij***_ are randomly computed in the area defined by its radius (see Fig. 6(a)). Additionally, for each pair of trial solutions (***s***_***ij***_ and ***s***_***ik***_), the middle point (***Mid***(***s***_***ij***_, ***s***_***ik***_)) of the segment connecting the pair is computed (see Fig. 6(b)). Then, the objective function value of the extreme points (*f*(***s***_***ij***_) and *f*(***s***_***ik***_)), as well as the middle point (*f*(***Mid***(***s***_***ij***_, ***s***_***ik***_))), is computed. If any objective function value of these new generated points is better of the original solution ***s***_***i***_, it will be updated, i.e., the centre of that subarea ***s***_***i***_ will be the one with the best objective function value. Additionally, if the objective function value in the middle solution is better than that of the extreme points, it may mean that it is in a hill (see Fig. 6(b)), so that it is considered a candidate to be included in the population list. On the contrary, the endpoints will be inserted as new poses. The radius of the new pose in the population will be that one associated with the current iteration. Figure 6(c) shows a summary of the whole process by keeping the references to the names in Fig. 6(a,b).

#### Replacement method

After the reproduction method has been applied, it is highly probable that the size of the population will be greater than the population size given by the input parameter *M*. Therefore, a mechanism for selecting the surviving solutions must be applied. Different types of replacements exist but, in this work, a deterministic and highly elitist one has been implemented: the original population and their corresponding offspring are grouped in an intermediate population, and then the *M* best solutions, i.e., the best poses, are selected as members of the population. The remaining ones are eliminated.

The implementation of this direct replacement involves the use of a sorting procedure whereby the poses are sorted according to their shape similarity value.

#### Improvement method

In order to introduce some noise into the search process, and hence avoid the convergence to local optima, a mutation operator is usually applied to the new offspring. Then, in most evolutionary or genetic algorithms, mutation mechanisms are included in the optimization procedure, which runs small random changes to the new individuals. However, for the present problem, the use of improvement methods has better shown to better approximate the poses towards the optima.

The improvement method implemented in OptiPharm is the local search method SASS, initially proposed by Solis and Wets^38^. It has been chosen mainly because it is a derivative-free optimization algorithm that can be applied to maximize any arbitrary function over a bounded subset of ${{\mathbb{R}}}^{N}$.

Several modifications have been included to adapt SASS to the problem at hand. In the following they are briefly described.

Algorithm SASS internally assumes that the range in which each variable is allowed to vary is the interval $[0,1]$. Since this is not our case, when necessary we use a function to rescale (normalize) the variable values to the interval $[0,1]$, and the function *denorm* to invert this process. In SASS, the new points are generated using a Gaussian perturbation $\xi \in {{\mathbb{R}}}^{3}$ over the search point (*x*,*α*) and a normalized bias term $b\in {{\mathbb{R}}}^{3}$ to direct the search. The standard deviation *σ* specifies the size of the sphere that most likely contains the perturbation vector. In this work, its upper bound *σ*_*ub*_ should have the same value as the normalized radius of the caller solution. Then, the parameter *σ*_*ub*_ is also considered an argument of SASS. Hence, any single step taken by the optimizer is no longer than the radius of the calling candidate solution. Finally, the stopping rules are determined by a maximum number of function evaluations (*fe*_*max*_) and by the maximum number of consecutive failures (*Maxfcnt*).

**Figure 7:**
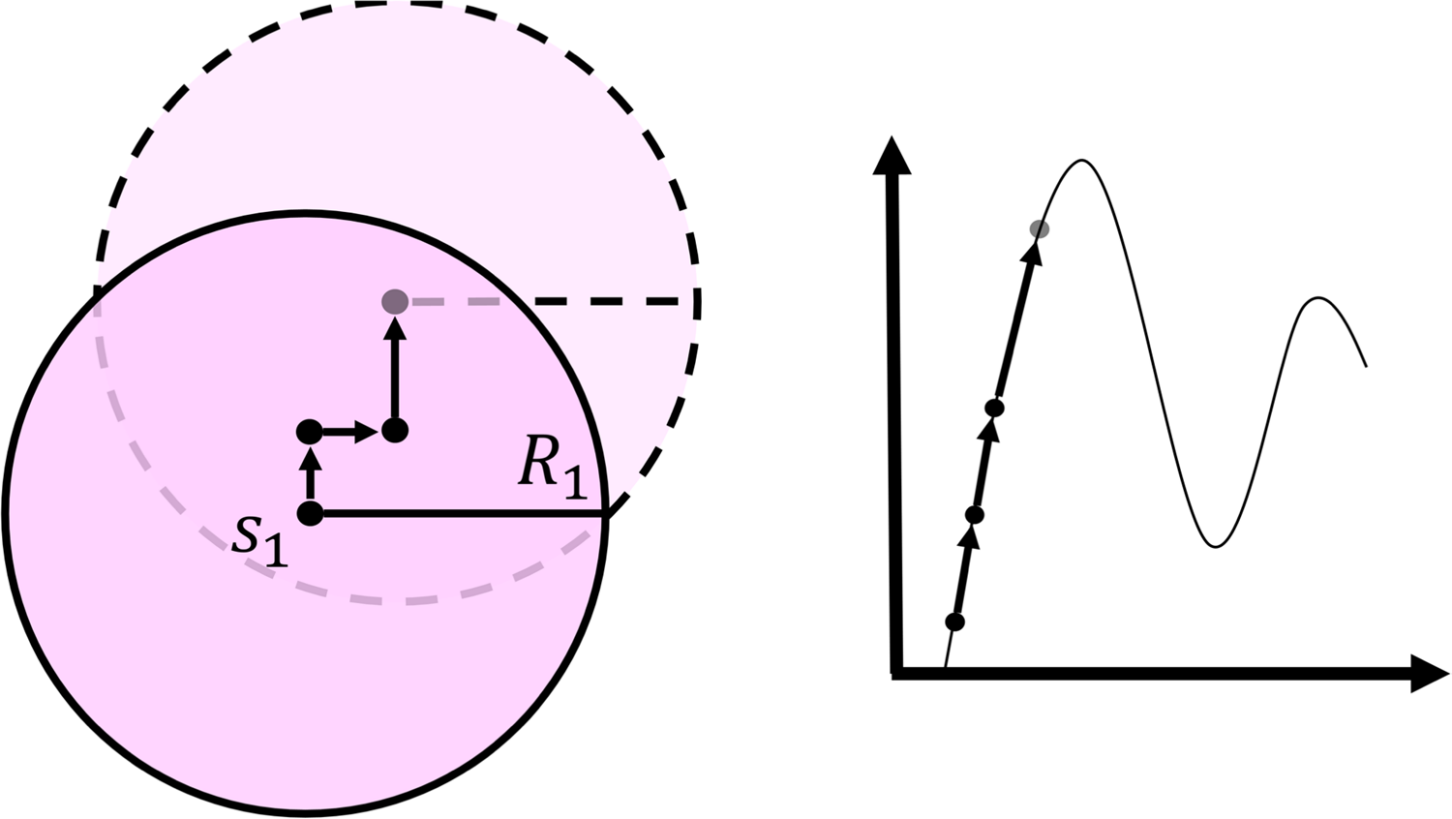
Example. The local optimizer SASS has been used as Improvement method. This figure shows the performance of SASS for a 2D case. SASS is a derivative-free optimization algorithm that can be applied to maximize an arbitrary function over a bounded subset of ${{\mathbb{R}}}^{N}$. It looks for an improving direction and moves the starting point along it by making changes of different sizes (if the number of consecutive successes is larger than a pre-specified value, then the advance along the suggested searching direction will be longer; otherwise, the size of the step will be reduced. The area of action of the optimizer is limited by the corresponding radius. In OptiPharm, the stopping rule of SASS is determined by a maximum number of function evaluations and by the maximum number of consecutive failures.

OptiPharm applies SASS to every pose in the population. See Fig. 7 for an illustrative example of its performance.

## Computational Experiments Framework

### Hardware setup

All the experiments carried out in this work have been executed in a Bullx R424-E3, which consists of 2 Intel Xeon E5 2650v2 (16 cores), 128 GB of RAM memory and 1 TB HDD.

### Methodology to test the performance of the algorithms

OptiPharm is a computer program which implements an evolutionary optimization algorithm which includes randomness in the search procedure. Then, in order to test its performance, we run each particular instance several times and we provide some statistical metrics, as usually is done when testing any heuristic algorithm in works in literature^39–43^. From a statistic point of view, a minimum number of 30 samples need to be considered for this^44^. Nevertheless, in this work, each particular instance has been run 100 times to increase confidence in the results. Then, figures as the average value and the standard deviation are computed to analyze its effectiveness and efficiency. It is important to highlight that executing several times a particular instance is only a methodology to analyze the robustness of the algorithm, but in the real world scenario, OptiPharm only needs a single run to provide reliable results.

Regarding WEGA, it is only run once for each particular instance, since it is deterministic (it uses a descent gradient method) and different executions always produce the same result.

### Benchmarks

Unlike OptiPharm, WEGA does not consider the hydrogen atoms in the shape similarity calculations. To be able to compare the results provided by both algorithms, OptiPharm has been configured to omit the hydrogens when computing the shape similarity score. Additionally, as WEGA does^27^, all the heavy atom radii have been set to 1.7 Å. Furthermore, all compound pairs are centred and aligned in the same way. Consequently, the molecule centroids have been located at the coordinates centre of the search space. Finally, each molecule has been aligned in such a way that its longest axis has been oriented at X-axis and the shortest along the Z-axis.

The underlying OptiPharm algorithm is parameterizable, which means that it can be fine-tuned depending on the user’s preferences. So users may prefer to obtain high-quality solutions at the expense of slightly increasing the computational effort, while others may want an acceptable solution with reasonable computing time. In this work, the parameters that control OptiPharm were tuned by trying several combinations of parameter values with a reduced set of problems, and following the guidelines described in a previous work^37^. As a consequence, two different sets of input parameters are proposed, given rise to two versions of OptiPharm with different aims:

(i) *OptiPharm Robust* (OpR). In this case, the set of input parameters is chosen to make OptiPharm reliable and robust; in other words, to allow OptiPharm to deeply explore and exploit the search space in the search for the best possible pose. In particular, the following values were considered: *N* = 200000 function evaluations, *M* = 5 starting poses, *t*_*max*_ = 5 iterations and ${R}_{{t}_{max}}=1$ as the smallest possible radius.
(ii) *OptiPharm Fast* (OpF). On this occasion, the parameters are tuned so that the running times are lower or similar to those of WEGA, enabling a fair comparison between both algorithms. The following values were considered: *N* = 1000 function evaluations, *M* = 5 starting poses, *t*_*max*_ = 5 iterations and a minimum radius of ${R}_{{t}_{max}}=5$.

From the previous paragraphs, one could infer that the number of starting poses, *M* = 5, and the number of iterations, *t*_*max*_ = 5, can be fixed independently of the goal pursued, while the smallest radius ${R}_{{t}_{max}}$, and most importantly, the number of function evaluations *N* have a bigger influence in both the effectiveness and the efficiency of the algorithm.

Four computational studies were designed by considering the well-known Food and Drug Administration (FDA)^45^, Directory of Useful Decoys (DUD)^46^, Directory of Useful Decoys - Enhanced (DUD-E)^47^ and Maybridge datasets. In the following sections, they are briefly described.

#### FDA

**Figure 8:**
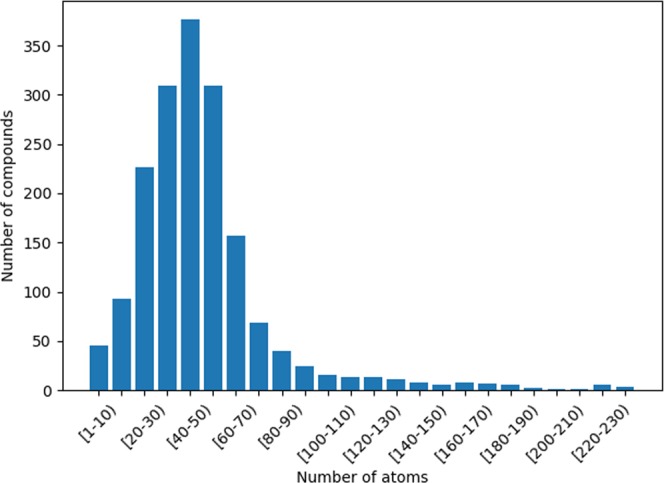
Number of compounds included on the FDA database, according to their number of atoms.

The FDA, a federal agency of the United States Department of Health and Human Services, is responsible for protecting and promoting public health by controlling, among other things, prescription and over-the-counter pharmaceutical drugs (medications). This agency provides a data set containing 1751 molecules, which represents approved medicines that can be used with safety in humans in the USA. It is a common practice^48^, in the current scenario, to identify which compound pairs in the FDA database share a high degree of shape similarity. To compare the performance of both OptiPharm and WEGA, a set of 40 query compounds were randomly selected from this database. In order to obtain a representative set of samples, the FDA dataset was initially sorted according to the number of atoms of the compounds, and divided into 24 intervals (see Fig. 8). Then, a subset of compounds was randomly chosen for each interval. The number of selected samples in each interval was proportional to the number of compounds it included.

#### DUD

Tests were also carried out applying shape similarity calculations and using different sets of molecules that are known to be active or inactive, and standard VS benchmark tests, such as the DUD^46^, whereby VS methods check how efficient they are at differentiating ligands that are known to bind to a given protein target, from non-binders or decoys. Input data for each molecule of each set contain its molecular structure and information about whether it is active or not. Information about active molecules for each protein of the DUD set was taken from experimental data. Decoys were prepared in order to resemble active ligands physically, but at the same time, to be chemically different from active molecules, making it very unlikely that they would act as binders. On average, for each ligand it is possible to find 36 decoy molecules that are very similar in physical terms, but with a very different topology. Details about how decoys were prepared (selected from already existing molecules in the ZINC database) can be found in the literature^46^, so that we shall only mention here the principal details required to understand the present study.

1. The initial database was built using 3.5 million Lipinski-compliant molecules from the ZINC database of commercially available compounds (version 6, December 2005).
2. Feature key fingerprints were calculated using the default type 2 substructure keys of CACTVS^49^ and the fingerprint-based similarity analysis was performed with the program SUBSET. Compounds with *Tc* values lower than 0.9 to any annotated ligand (named as actives) were selected. This reduced the number of ZINC compounds to 1.5 million molecules topologically dissimilar to the ligands.
3. The program QikProp (Schrodinger, LLC, New York, NY) was used to calculate 32 physical properties of all the annotated ligands and selected ZINC compounds from the previous step, and QikSim (Schrodinger, LLC, New York, NY) was applied to prioritize ZINC compounds possessing similar physical properties to any of the ligands.
4. A weight of 4 was used to emphasize the druglike descriptors (molecular weight, number of hydrogen bond acceptors, number of hydrogen bond donors, number of rotatable bonds, and log P), while the rest of the descriptors were ignored (weight 0) during the similarity analysis procedure.
5. Finally, thirty-six decoy compounds were selected for each ligand, leading to a total of 95316 decoys that were similar in terms of physical properties but topologically dissimilar to the 2950 annotated ligands. The total number of decoys is less than 36 times the number of annotated ligands because some ligands had the same decoys.

The original DUD database downloaded from http://zinc.docking.org has been used.

#### DUD-E

The DUD-E^47^ is a well-known benchmark for structure-based virtual screening methods from the Shoichet Lab at UCSF^47^. The methodology of the DUDE benchmark is fully described in its original work^47^. Briefly, the benchmark is constructed by first gathering diverse sets of active molecules for a set of target proteins. Analogue bias is mitigated by removing similar actives; similar actives are eliminated by first clustering the actives based on scaffold similarity, then selecting exemplar actives from each cluster. Then, each active molecule is paired with a set of property-matched decoys (PMD)^50^. PMD are selected to be similar to each other and to known actives with respect to some 1-dimensional physicochemical descriptors (e.g., molecular weight) while being topologically dissimilar based on some 2D fingerprints (e.g., ECFP^51^). The enforcement of the topological dissimilarity supports the assumption that the decoys are likely to be inactive because they are chemically different from any know active. The benchmark consists of 102 targets, 22,886 actives (an average of 224 actives per target) and 50 PMD per active^52^. The original DUD-E database downloaded from http://dude.docking.org/ has been used in this work.

#### Maybridge

Maybridge^53^ Screening Hit Discovery collection (over 53,000 compounds) is a commercial library of small hit-like and lead-like organic compounds of high diversity (Tanimoto Clustering at 0.9)^54^, that covers ca. 87% of the 400,000 theoretical drug pharmacophores with general compliance with the Lipinsky rule of five and of good ADMET properties. The HitCreatorTM Collection (selection of 14,400 of Maybridge screening compounds) aims to represent the diversity of the main collection covering the drug-like chemical space. Maybridge also offers a fragment library (30,000 fragments), a hit-to-lead building block collection, and a Ro3 2500 diversity fragment library (2500 fragments) with a Tanimoto similarity index of 0.66 (based on standard Daylight fingerprinting), assured solubility, optimized for SPR and Ro3 compliant. It provides special collections of Fluoro^55^, Fluoro and Bromo-fragment libraries^56^. The original Maybridge database downloaded from https://www.maybridge.com has been used in this study.

#### The AUC metric

In this work, to measure the goodness of the algorithms when distinguishing between ligands and decoys, the Area Under a ROC Curve (AUC) was computed, as previously done in other related papers^27^. See^57^ for an in-depth description of calculation. Broadly speaking, the AUC of a set of elements is computed by considering a descriptor value that is associated to each element.

For the problem at hand, such a descriptor is given by Equation 4, which measures the shape similarity between two molecules, A and B. However, before computing the AUC, given a query molecule and a set of molecules the similarity to which is to be computed, a optimization problem must be solved to obtain the shape similarity scores for each molecule in the set. Then, the list is sorted in descending order according to the shape similarity values. Without going into detail, an AUC value equal to 1 means that such a particular algorithm has been able to differentiate perfectly between two datasets - in our case, between ligands and decoys. In other words, it is possible to determine a cut-off point (a real value) which divides the list into two intervals that contain all the decoys and ligands, respectively. When it is not possible to determine only two intervals, more cut-off points should be considered in an incremental way. Of course, the larger the number of intervals, the smaller the AUC value. However, AUC values smaller than or equal to 0.5 mean the algorithm has poor effectiveness, i.e., a random method would have achieved a similar classification.

## Results and Discussion

### Results obtained for FDA database

It is important to mention that for all the algorithms and all the instances, a score equal to 1 is obtained when a molecule is compared to itself. Thus, from here on, when we mention “the molecule with the highest shape similarity to a query compound”, and noted by BestComp, we exclude the case where target molecule and query are equal.

Table 1 shows, for each query compound, its number of atoms (nA), the other compound from the FDA database with the highest shape similarity (BestComp) and the associated function score (Tc), according to OpR, OpF and WEGA. As can be seen, the OpR algorithm provides the highest shape similarity values Tc, although it is also the most time-consuming method according to Table 2. This means that better predictions can be accomplished by using OpR when there are no time constraints. However, if lower execution times are required, algorithms such as OpF or WEGA should be considered.

**Table 1:** Results obtained for 40 query compounds from the FDA database.

query		OpR		OpF			WEGA		
name	nA	BestComp	Tc	BestComp	Tc	(i, Tc(OpR))	BestComp	Tc	(i, Tc(OpR))
DB00529	7	DB00828	0.921	DB00828	0.920	—	DB00828	0.921	—
DB00331	9	DB01189	0.940	DB01189	0.936	—	DB01189	0.940	—
DB01365	12	DB00191	0.944	DB00191	0.943	—	DB00191	0.944	—
DB01352	15	DB00306	0.891	DB00306	0.884	—	DB00237	0.872	(2, 0.872)
DB00380	19	DB00816	0.842	DB00816	0.822	—	DB00816	0.842	—
DB06216	20	DB00370	0.905	DB00370	0.902	—	DB09304	0.856	(2, 0.869)
DB00674	21	DB01619	0.865	DB01619	0.855	—	DB00370	0.850	(2, 0.850)
DB00632	23	DB00464	0.724	DB00464	0.719	—	DB00464	0.717	—
DB07615	24	DB01250	0.799	DB01250	0.797	—	DB01250	0.799	—
DB00693	25	DB01619	0.841	DB01619	0.793	—	DB01068	0.825	(2, 0.825)
DB00887	25	DB06614	0.745	DB06614	0.732	—	DB04938	0.733	(2, 0.730)
DB09219	25	DB00434	0.819	DB00792	0.805	(3, 0.812)	DB00792	0.812	(3, 0.812)
DB00351	27	DB04839	0.941	DB04839	0.936	—	DB00603	0.902	(2, 0.902)
DB00381	28	DB01023	0.819	DB01023	0.732	—	DB06712	0.707	(5, 0.706)
DB09237	28	DB01054	0.717	DB01054	0.648	—	DB01115	0.686	(4, 0.685)
DB01198	29	DB00402	0.933	DB00402	0.929	—	DB00402	0.933	—
DB00876	30	DB09039	0.664	DB05239	0.651	(3, 0.653)	DB05239	0.653	(3, 0.653)
DB01621	32	DB01148	0.694	DB01148	0.693	—	DB01148	0.694	—
DB09236	33	DB00270	0.672	DB01115	0.615	(2, 0.669)	DB01433	0.662	(3, 0.662)
DB08903	37	DB00333	0.653	DB00333	0.610	—	DB06703	0.630	(4, 0.630)
DB00728	38	DB01339	0.820	DB01339	0.816	—	DB01339	0.820	—
DB01419	42	DB06605	0.630	DB06605	0.626	—	DB06605	0.630	—
DB00320	43	DB01413	0.629	DB01413	0.618	—	DB01413	0.629	—
DB01232	49	DB01082	0.549	DB01082	0.535	—	DB01082	0.549	—
DB00246	50	DB01261	0.761	DB01261	0.738	—	DB01261	0.761	—
DB00503	50	DB00845	0.499	DB01319	0.461	(4, 0.496)	DB01319	0.498	(4, 0.496)
DB09114	50	DB08993	0.476	DB04894	0.411	(6, 0.416)	DB08993	0.477	—
DB00254	55	DB00595	0.877	DB00595	0.874	—	DB00595	0.877	—
DB00309	55	DB00541	0.634	DB00541	0.618	—	DB00541	0.634	—
DB06439	57	DB00207	0.515	DB00207	0.494	—	DB00212	0.513	(2, 0.513)
DB01196	60	DB00286	0.784	DB00286	0.779	—	DB00286	0.784	—
DB01078	66	DB00511	0.502	DB00511	0.479	—	DB00511	0.503	—
DB01590	68	DB00877	0.469	DB00385	0.459	(2, 0.464)	DB00877	0.469	—
DB04894	80	DB00364	0.482	DB00364	0.468	—	DB00864	0.453	(3, 0.453)
DB04786	86	DB01078	0.387	DB09158	0.306	(3, 0.369)	DB01078	0.387	—
DB00732	87	DB01045	0.434	DB01045	0.417	—	DB01045	0.434	—
DB00403	94	DB00035	0.394	DB06402	0.355	(4, 0.376)	DB08874	0.386	(2, 0.386)
DB00050	102	DB00569	0.396	DB00569	0.391	—	DB00569	0.396	—
DB06699	117	DB00091	0.454	DB00512	0.409	(2, 0.414)	DB09099	0.412	(3, 0.411)
DB06219	128	DB00512	0.422	DB00364	0.354	(2, 0.409)	DB00364	0.410	(2, 0.409)

For each query, its nA and the BestComp with the highest Tc is shown, according to OpR, OpF and WEGA. Note that the score Tc is equal to 1 when the query compound is compared with itself for all the instances and algorithms, so that BestComp really represents the second most similar molecule to the query.

**Table 2:** Performance results obtained by the different similarity methods.

query	nA	OpR		OpF		WEGA	speedup
		Av	SD	Av	SD	T	
DB00529	7	61.2	0.560	4.8	0.008	16.4	3.4
DB00331	9	77.4	0.752	5.8	0.041	17.5	3.0
DB01365	12	96.7	0.714	7.3	0.004	16.9	2.3
DB01352	15	116.5	0.823	9.1	0.037	19.5	2.1
DB00380	19	165.1	1.425	11.0	0.028	20.4	1.9
DB06216	20	169.2	1.203	11.8	0.030	25.3	2.1
DB00674	21	169.9	1.123	12.3	0.011	20.6	1.7
DB00632	23	130.4	1.564	11.3	0.005	22.3	2.0
DB07615	24	205.4	1.385	13.4	0.010	22.4	1.7
DB00693	25	215.2	2.158	14.5	0.017	24.2	1.7
DB00887	25	213.5	1.547	14.2	0.001	21.6	1.5
DB09219	25	223.1	1.709	14.3	0.010	22.6	1.6
DB00351	27	220.7	1.980	15.3	0.017	23.4	1.5
DB00381	28	227.5	1.499	15.5	0.013	32.1	2.1
DB09237	28	227.4	1.222	15.8	0.001	22.8	1.4
DB01198	29	223.9	1.354	14.6	0.000	23.1	1.6
DB00876	30	262.0	1.878	17.1	0.002	23.7	1.4
DB01621	32	267.1	1.475	17.2	0.017	24.7	1.4
DB09236	33	280.7	2.230	18.1	0.059	27.0	1.5
DB08903	37	289.3	2.188	20.0	0.045	25.5	1.3
DB00728	38	284.6	1.787	20.3	0.032	25.8	1.3
DB01419	42	359.2	2.371	21.7	0.031	28.5	1.3
DB00320	43	355.6	2.374	22.8	0.016	25.6	1.1
DB01232	49	395.7	2.896	25.7	0.036	29.2	1.1
DB00246	50	250.7	1.719	15.5	0.073	22.6	1.5
DB00503	50	416.6	2.743	26.3	0.008	31.0	1.2
DB09114	50	388.3	2.782	23.9	0.005	31.6	1.3
DB00254	55	263.1	1.919	17.4	0.003	25.3	1.5
DB00309	55	377.3	2.626	28.5	0.022	30.8	1.1
DB06439	57	434.8	2.937	29.3	0.048	32.9	1.1
DB01196	60	244.7	1.599	15.9	0.054	27.1	1.7
DB01078	66	485.9	3.538	28.9	0.050	36.0	1.2
DB01590	68	495.1	3.297	31.9	0.010	39.4	1.2
DB04894	80	550.4	3.876	37.8	0.006	40.7	1.1
DB04786	86	598.7	4.728	32.3	0.002	45.4	1.4
DB00732	87	628.5	4.147	40.1	0.015	44.2	1.1
DB00403	94	609.5	5.072	39.6	0.041	49.5	1.3
DB00050	102	664.1	4.834	45.5	0.050	51.3	1.1
DB06699	117	725.6	5.257	50.8	0.005	55.0	1.1
DB06219	128	828.4	7.030	52.0	0.090	63.4	1.2
mean	46	330.0	2.408	21.7	0.024	29.7	1.6

Columns represent: DrugBank code for each molecule, its corresponding nA, average running time (in seconds) and standard deviation obtained by OpR and OpF (see columns 3–6), execution time spent by WEGA (see column 7), and speedup of OpF against WEGA.

To the best of our knowledge, no algorithm, method or program exists that is able to provide with certainty the most similar molecule to a given query compound. Until this work, WEGA was the algorithm providing the most optimal shape similarity values^27,34^. Now, as can be seen in Table 1, OpR improves on WEGA in terms of the ability to find higher values of shape similarity when processing a query compound against a ligand database. Therefore, to analyze the effectiveness of OpF and WEGA in term of their predictions, the solutions provided by OpR will be considered the optimal ones.

As can be seen in Table 1, the predictions of WEGA coincide with those of OpR in 22 out of 40 cases, while OpF does it in 30 out of 40 occasions. This represents a small advantage to OpF against WEGA in terms of success in the predictions. Additionally, from Table 2, which shows the computing times, one can appreciate that OpF is quicker than WEGA.

Furthermore, it is important to study the instances where the predictions of OpF and WEGA do not coincide with those achieved by OpR. This occurs in 18 out of 40 cases for WEGA, and 10 times for OpF. Then, for each particular query, the 1751 compounds are sorted in descending order according to the shape similarity value obtained by OpR. Next, it is computed the position *i* in the list where the BestComp achieved by OpF (resp. WEGA) is, and which one shape similarity value, Tc(OpR). This information is shown in Table 1, columns 6 and 9 for OpF and WEGA, respectively. Broadly speaking, in most of cases the predictions carried out by OpF are located in a better position in the OpR list than the predictions proposed by WEGA.

**Figure 9:**
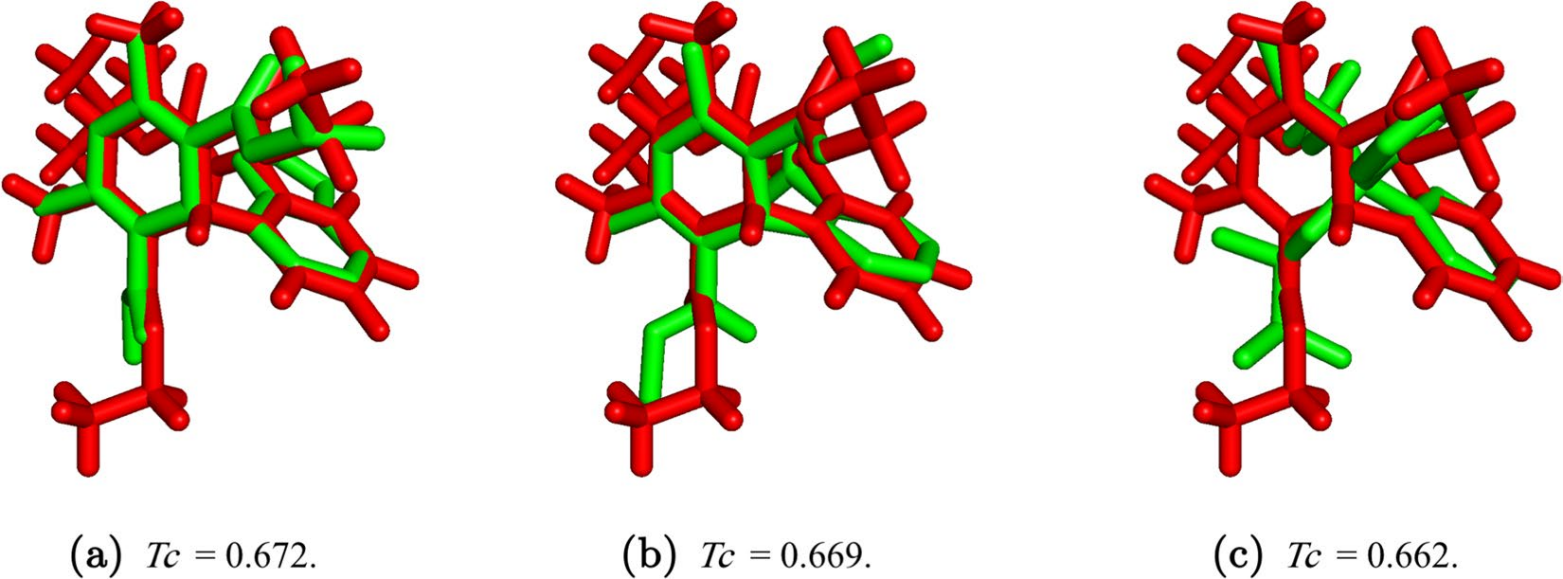
Depiction of shape similarity between the query DB09236 and (**a**) the molecule DB00270, (**b**) the compound DB01115, and (**c**) the molecule DB01433, when they are optimized by OpR.

It is important to mention that, in general, OptiPharm is designed to maintain population diversity and to investigate many promising poses in parallel, avoiding the genetic drift towards a single (local or global) optimal pose. However, depending on the selected set of parameters, the accuracy when approximating to the optima may be higher or lower. For this reason, OpF has been fine-tuned to explore the search space looking for the most promising poses, but without wasting time by “polishing” them. In optimization terms, the input parameters are selected to determine the highest peaks in the search space, but not to actually reach the top of the highest peak. Even when OpF proposes as BestComp the same compound as OpR (or even WEGA), its shape similarity value may be smaller. If the algorithm is allowed to run longer, as with OpR, the identified poses can be polished, increasing the score value. In this case we prioritize the computational effort. Figure 9 depicts a graphical example of this fact, specifically the query DB09236 from the FDA database, whose result can be seen in Table 1. Considering this query, OpR reveals that DB00270 is the compound which maximizes the shape similarity function, with a score value equal to *Tc* = 0.672 (see Fig. 9(a)). OpF reveals that molecule DB01115 maximises the shape similarity function with a score value equal to *Tc* = 0.615. Finally, WEGA reveals that the molecule DB01433 maximizes the shape similarity function with *Tc* = 0.662. Apparently, WEGA achieves a more similar compound than OpF, since it provides as solution a compound with a higher score than the one proposed by OpF. However, when OpR optimizes the query with the molecule DB01115 proposed by OpF, it provides a score value of 0.669 (see Fig. 9(b)). By contrast, OpR gives a value of 0.662 when it optimizes the query with the compound DB01433 given by WEGA, (see Fig. 9(c)). This means that the solution provided by OpF is more similar in terms of shape than that of WEGA.

Table 2 shows performance values among the different methods. Clearly, the slowest algorithm is OpR, since it has been fine-tuned to be robust and accurate. Even so, the time values are not extremely high when compared against the other two methods. In fact, taking into account the possibility of using high-performance computing to accelerate it (please, see Future Work Section), it would be perfectly justifiable to use Robust mode to increase the percentage success in the predictions. For its part, OpF is the fastest algorithm, reducing on average the computational effort of WEGA almost 3.5 times. Besides, as can be appreciated in the Speedup column, the lower the number of atoms, the greater the increase in speed obtained by OpF. Additionally, it is important to mention that OpF is able to adapt itself to the complexity of the problem to solve.

Finally, it is interesting to remark that, in spite of the randomness included at some stages of the OptiPharm algorithm, its variability is almost negligible, as can be appreciated from the standard deviation values provided in Table 2.

### Results obtained for DUD and DUD-E databases

Tables 3 and 4 show the results of testing the shape-based VS performance of both OptiPharm (in its two versions) and WEGA against the DUD and DUD-E databases, respectively. Metrics of AUC values and execution time have been computed. As previously was mentioned, to test the OptiPharm reliability, each particular instance has been run 100 times and average values have been computed. Furthermore, the corresponding SD has also been provided. Regarding WEGA, since it is deterministic, only one single execution has been carried out for each particular instance and the corresponding values have been shown.

**Table 3:** DUD database.

name	AUC					Time				
	OpR		OpF		WEGA	OpR		OpF		WEGA
	Av	SD	Av	SD	AUC	Av	SD	Av	SD	Time
ace	0.39	0.013	0.44	0.021	0.33	278.7	0.046	15.2	0.000	31.0
ache	0.71	0.004	0.71	0.008	0.72	645.5	0.059	35.5	0.003	67.0
ada	0.67	0.003	0.71	0.011	0.66	67.8	0.011	4.9	0.000	12.5
alr2	0.24	0.003	0.28	0.012	0.22	87.3	0.015	6.8	0.000	13.9
ampc	0.70	0.005	0.75	0.020	0.71	68.6	0.013	5.0	0.000	10.9
ar	0.73	0.003	0.73	0.005	0.72	209.2	0.020	18.1	0.001	41.2
cdk2	0.60	0.010	0.58	0.010	0.59	184.3	0.026	12.4	0.000	28.7
comt	0.43	0.017	0.45	0.016	0.37	45.6	0.007	3.3	0.000	10.0
cox1	0.49	0.003	0.51	0.009	0.48	57.2	0.009	4.7	0.000	12.6
cox2	0.95	0.002	0.93	0.004	0.95	1738.5	0.112	109.6	0.006	1038.6
dhfr	0.65	0.003	0.61	0.007	0.65	1392.8	0.081	83.6	0.006	742.6
egfr	0.59	0.003	0.54	0.006	0.57	2128.5	0.100	137.3	0.008	962.1
er_agonist	0.79	0.003	0.80	0.007	0.79	228.4	0.026	17.6	0.001	120.7
er_antagonist	0.73	0.008	0.73	0.015	0.72	262.4	0.029	15.2	0.000	70.0
fgfr1	0.41	0.001	0.45	0.003	0.40	668.7	0.047	39.4	0.003	387.6
fxa	0.60	0.007	0.60	0.010	0.68	1161.2	0.073	65.5	0.005	244.6
gart	0.31	0.007	0.41	0.012	0.27	197.0	0.024	11.6	0.000	49.6
gpb	0.85	0.004	0.82	0.008	0.84	128.9	0.016	10.5	0.000	35.5
gr	0.62	0.005	0.66	0.008	0.62	365.2	0.034	27.4	0.002	53.5
hivpr	0.78	0.011	0.71	0.011	0.76	622.7	0.063	36.0	0.001	51.1
hivrt	0.75	0.011	0.75	0.010	0.75	143.8	0.019	9.8	0.000	34.0
hmga	0.75	0.012	0.75	0.015	0.77	240.7	0.027	14.9	0.000	78.5
hsp90	0.68	0.009	0.77	0.016	0.66	128.7	0.019	8.2	0.000	30.5
inha	0.61	0.007	0.53	0.009	0.60	479.7	0.045	32.4	0.002	84.4
mr	0.84	0.004	0.84	0.007	0.84	66.6	0.011	5.6	0.000	10.7
na	0.86	0.008	0.83	0.008	0.85	165.4	0.017	12.4	0.000	31.6
p38	0.50	0.003	0.45	0.012	0.47	1997.2	0.125	112.7	0.006	371.6
parp	0.50	0.003	0.46	0.008	0.49	96.3	0.016	7.8	0.000	33.8
pde5	0.75	0.008	0.74	0.009	0.75	420.6	0.038	23.5	0.001	124.9
pdgfrb	0.45	0.003	0.47	0.006	0.46	964.0	0.058	54.3	0.005	145.7
pnp	0.61	0.008	0.61	0.020	0.63	71.4	0.011	5.6	0.000	17.2
ppar_gamma	0.68	0.014	0.72	0.011	0.70	1055.6	0.086	50.2	0.003	134.2
pr	0.62	0.018	0.65	0.029	0.61	151.7	0.024	10.9	0.000	44.5
rxr_alpha	0.90	0.023	0.91	0.013	0.91	122.0	0.016	7.3	0.000	13.8
sahh	0.89	0.006	0.87	0.007	0.89	87.9	0.012	6.7	0.000	19.5
src	0.32	0.003	0.38	0.008	0.30	1388.0	0.072	74.3	0.006	272.7
thrombin	0.50	0.009	0.57	0.013	0.55	510.2	0.045	28.6	0.001	145.4
tk	0.56	0.018	0.56	0.017	0.58	47.8	0.008	4.2	0.000	20.6
trypsin	0.28	0.006	0.33	0.009	0.26	255.4	0.024	12.6	0.000	41.0
vegfr2	0.61	0.006	0.60	0.008	0.61	323.5	0.027	21.2	0.001	49.5
mean	0.62	0.007	0.63	0.011	0.61	481.4	0.038	29.1	0.002	142.2

For each query compound, the average AUC value and the mean running time (in seconds) over 100 independent executions were computed with both OpR and OpF. For the sake of completeness, the SD is also provided for both OpR and OpF versions. WEGA is a deterministic algorithm, so it was only executed once and its computed AUC value and the execution time are included. The last row of the table shows average values for the query molecules.

**Table 4:** DUD-E database.

name	AUC					Time				
	OpR		OpF		WEGA	OpR		OpF		WEGA
	Av	SD	Av	SD	AUC	Av	SD	Av	SD	Time
aa2ar	0.57	0.000	0.56	0.011	0.57	2656.8	24.748	45.6	1.125	1363.1
abl1	0.52	0.001	0.53	0.003	0.52	905.6	5.756	35.8	1.197	430.4
ace	0.51	0.000	0.50	0.015	0.53	1392.5	4.369	27.8	0.827	710.6
aces	0.24	0.000	0.27	0.006	0.24	1733.6	3.072	26.9	0.901	978.7
ada	0.63	0.000	0.58	0.041	0.71	245.6	1.570	5.3	0.193	232.0
ada17	0.48	0.000	0.47	0.002	0.48	1894.6	3.297	47.6	1.796	1011.2
adrb1	0.36	0.002	0.33	0.003	0.36	966.5	1.987	32.6	1.002	634.5
adrb2	0.37	0.001	0.37	0.003	0.38	1155.9	10.912	19.8	0.736	555.6
akt1	0.26	0.001	0.26	0.008	0.26	1062.5	1.992	40.4	0.977	675.6
akt2	0.41	0.001	0.34	0.004	0.39	504.1	5.271	17.0	0.563	210.9
aldr	0.54	0.001	0.50	0.007	0.54	565.1	2.701	23.0	0.789	310.2
ampc	0.63	0.000	0.52	0.007	0.64	118.9	1.736	2.5	0.096	101.8
andr	0.63	0.000	0.60	0.002	0.63	595.8	17.953	26.1	0.774	398.3
aofb	0.44	0.000	0.45	0.002	0.44	135.2	0.411	3.1	0.090	198.3
bace1	0.53	0.000	0.46	0.018	0.54	1284.8	4.041	37.8	1.197	758.4
braf	0.56	0.002	0.48	0.005	0.55	766.7	9.025	14.8	0.424	352.3
cah2	0.45	0.003	0.44	0.002	0.44	765.5	13.342	37.6	1.297	1173.8
casp3	0.41	0.000	0.44	0.001	0.39	561.4	0.763	13.8	0.384	436.6
cdk2	0.66	0.001	0.64	0.004	0.66	3007.8	71.465	70.7	1.907	837.4
comt	0.60	0.002	0.56	0.005	0.62	122.9	1.591	5.3	0.146	111.7
cp2c9	0.43	0.000	0.43	0.005	0.44	408.3	2.055	11.1	0.326	280.0
cp3a4	0.53	0.001	0.53	0.007	0.53	1370.1	6.526	32.5	0.904	430.5
csf1r	0.55	0.000	0.58	0.006	0.60	1085.7	37.667	27.9	0.806	397.4
cxcr4	0.71	0.002	0.65	0.003	0.73	231.4	1.851	4.0	0.117	112.3
def	0.69	0.000	0.55	0.008	0.69	324.2	15.676	6.5	0.188	191.6
dhi1	0.64	0.000	0.67	0.002	0.64	1200.7	7.467	26.8	0.689	703.7
dpp4	0.57	0.000	0.55	0.002	0.57	3618.4	6.684	62.8	1.866	1402.7
drd3	0.30	0.000	0.29	0.002	0.29	2085.8	6.171	56.3	1.884	1174.4
dyr	0.40	0.001	0.38	0.004	0.40	976.6	26.652	63.6	1.888	624.9
egfr	0.52	0.001	0.45	0.005	0.54	3601.2	99.478	54.8	1.780	1460.8
esr1	0.64	0.000	0.64	0.003	0.63	1994.7	6.168	43.2	1.385	749.2
esr2	0.69	0.000	0.65	0.003	0.68	1300.2	2.196	25.8	0.919	693.1
fa7	0.66	0.001	0.60	0.003	0.52	2691.7	8.408	111.9	1.731	184.1
fa10	0.51	0.002	0.53	0.008	0.67	761.6	2.982	14.0	0.388	589.3
fabp4	0.69	0.009	0.62	0.010	0.67	285.8	3.176	11.1	0.425	119.0
fak1	0.69	0.002	0.67	0.002	0.67	648.8	6.135	37.6	0.863	160.3
fgfr1	0.47	0.002	0.47	0.004	0.46	50.5	0.788	1.7	0.052	50.2
fkb1a	0.68	0.001	0.67	0.008	0.72	458.1	16.792	10.4	0.308	253.5
fnta	0.55	0.000	0.47	0.004	0.55	6081.6	4.037	186.2	5.634	2102.5
fpps	0.86	0.001	0.81	0.002	0.88	250.7	1.818	8.7	0.267	221.3
gcr	0.52	0.000	0.48	0.002	0.50	1046.0	1.883	29.3	0.970	624.2
glcm	0.36	0.002	0.30	0.001	0.35	132.6	6.153	3.4	0.104	132.2
gria2	0.59	0.001	0.56	0.004	0.60	740.5	8.288	22.9	0.865	418.7
grik1	0.62	0.001	0.67	0.004	0.61	262.6	4.979	8.3	0.273	253.6
hdac2	0.34	0.000	0.31	0.002	0.35	521.0	2.440	10.6	0.354	400.5
hdac8	0.42	0.000	0.40	0.004	0.43	528.3	5.144	11.8	0.372	353.7
hivint	0.41	0.001	0.35	0.004	0.41	384.1	1.260	12.3	0.389	221.2
hivpr	0.70	0.001	0.69	0.009	0.71	4748.8	5.144	133.4	3.808	1354.1
hivrt	0.52	0.000	0.49	0.001	0.52	1107.6	4.250	42.3	1.469	573.8
hmdh	0.75	0.000	0.71	0.003	0.74	976.8	7.565	19.7	0.549	399.8
hs90a	0.63	0.001	0.60	0.008	0.64	390.3	8.946	9.7	0.189	183.7
hxk4	0.64	0.001	0.49	0.002	0.62	358.3	12.647	11.5	0.382	188.1
igf1r	0.48	0.002	0.46	0.004	0.50	1048.3	2.244	31.8	1.059	401.7
inha	0.39	0.002	0.34	0.005	0.43	130.6	0.407	2.3	0.075	79.6
ital	0.39	0.002	0.44	0.007	0.38	1157.5	2.996	30.1	0.827	459.8
jak2	0.68	0.000	0.64	0.004	0.68	412.2	4.734	8.1	0.277	283.3
kif11	0.83	0.000	0.58	0.006	0.83	606.8	6.053	8.2	0.272	318.1
kit	0.43	0.000	0.41	0.003	0.44	678.5	0.307	15.9	0.559	325.9
kith	0.69	0.003	0.65	0.002	0.70	153.3	1.227	3.7	0.145	104.5
kpcb	0.58	0.000	0.52	0.004	0.59	622.6	6.039	13.6	0.471	310.2
lck	0.46	0.001	0.43	0.002	0.44	2110.1	4.281	40.6	1.237	1121.1
lkha4	0.52	0.000	0.52	0.003	0.58	599.4	0.866	9.7	0.298	365.4
mapk2	0.65	0.000	0.61	0.003	0.65	376.4	1.190	10.4	0.316	210.0
mcr	0.64	0.000	0.59	0.002	0.63	292.4	3.096	6.1	0.200	175.4
met	0.68	0.002	0.73	0.007	0.72	2182.0	15.724	46.8	1.571	564.2
mk01	0.39	0.001	0.38	0.002	0.40	256.9	0.739	6.1	0.209	154.7
mk10	0.45	0.000	0.49	0.005	0.44	559.1	2.017	11.5	0.362	258.8
mk14	0.54	0.001	0.52	0.003	0.54	6277.2	25.295	139.1	4.452	1404.4
mmp13	0.56	0.000	0.55	0.002	0.60	3671.8	7.850	63.4	2.080	1525.6
mp2k1	0.42	0.000	0.53	0.003	0.45	722.9	17.182	11.7	0.383	339.2
nos1	0.35	0.001	0.33	0.003	0.35	366.6	8.322	6.3	0.197	267.2
nram	0.85	0.000	0.79	0.002	0.85	357.0	8.898	6.2	0.163	200.7
pa2ga	0.60	0.000	0.62	0.005	0.60	416.6	3.433	8.6	0.324	218.3
parp1	0.64	0.000	0.63	0.001	0.64	1481.6	2.181	43.2	1.314	981.3
pde5a	0.59	0.000	0.56	0.002	0.56	2777.0	56.574	37.4	1.222	1243.3
pgh1	0.70	0.000	0.72	0.004	0.71	620.4	1.826	15.3	0.412	425.2
pgh2	0.79	0.000	0.74	0.001	0.79	1130.5	2.544	35.5	1.161	791.9
plk1	0.53	0.000	0.47	0.006	0.54	797.5	6.744	11.3	0.346	267.3
pnph	0.74	0.000	0.70	0.001	0.74	264.4	1.521	5.9	0.185	212.8
ppara	0.76	0.000	0.75	0.003	0.77	2109.3	27.199	39.3	1.456	870.2
ppard	0.47	0.001	0.34	0.002	0.44	1557.1	2.223	31.7	0.856	503.6
pparg	0.45	0.001	0.43	0.002	0.45	2867.4	34.034	69.3	1.942	1122.2
prgr	0.72	0.001	0.69	0.002	0.71	1148.7	12.219	33.8	1.017	469.9
ptn1	0.31	0.001	0.29	0.005	0.30	348.1	1.425	8.9	0.264	290.6
pur2	0.37	0.000	0.26	0.009	0.33	242.8	1.844	4.9	0.153	146.9
pygm	0.58	0.000	0.62	0.005	0.57	241.4	1.812	5.9	0.162	173.2
pyrd	0.84	0.000	0.80	0.001	0.85	343.0	0.970	8.2	0.237	233.1
reni	0.59	0.002	0.56	0.003	0.58	970.5	21.357	39.6	1.241	292.4
rock1	0.55	0.000	0.52	0.002	0.54	216.7	3.338	4.3	0.167	207.4
rxra	0.61	0.000	0.49	0.003	0.60	410.0	10.792	8.5	0.312	258.6
sahh	0.87	0.000	0.60	0.003	0.86	123.9	0.394	2.1	0.105	131.8
src	0.55	0.002	0.53	0.002	0.60	4995.2	6.656	271.2	7.781	1318.6
tgfr1	0.60	0.001	0.49	0.003	0.59	514.3	9.723	10.5	0.373	350.7
thb	0.79	0.000	0.75	0.001	0.81	651.8	1.963	12.8	0.428	321.2
thrb	0.45	0.000	0.43	0.003	0.45	2427.1	114.339	71.5	2.444	1205.4
try1	0.57	0.000	0.56	0.001	0.57	2483.4	50.893	60.8	2.151	1123.2
tryb1	0.38	0.000	0.36	0.003	0.39	555.0	2.471	8.3	0.275	277.8
tysy	0.65	0.002	0.61	0.007	0.66	705.4	0.347	16.4	0.525	266.7
urok	0.40	0.000	0.40	0.002	0.41	511.1	1.834	13.8	0.466	342.5
vgfr2	0.57	0.000	0.60	0.003	0.59	1816.6	16.649	42.6	1.154	902.3
wee1	0.65	0.001	0.47	0.018	0.62	695.5	5.638	12.1	0.377	204.3
xiap	0.79	0.004	0.76	0.010	0.78	530.0	6.233	16.1	0.448	187.4
mean	0.56	0.001	0.53	0.005	0.56	1152.9	10.256	30.1	0.912	516.6

For each query compound, the average AUC value and the mean running time (in seconds) over 100 independent executions were computed with both OpR and OpF. For the sake of completeness, the standard deviation SD is also provided for both OpR and OpF versions. WEGA is a deterministic algorithm, so it was only executed once and its computed AUC value and the execution time are included. The last row of the table shows average values for the query molecules.

In general terms, the SD values obtained for OpR and OpF are quite small, which indicates that their variability is small, and that (i) they converge toward the same optima in spite of the included randomness and (ii) the computing time is practically the same when different executions of the same instance are carried out.

Focusing now on Table 3, it is possible to infer that the three algorithms are equivalent in terms of accuracy of the predictions, i.e. they obtain about the same AUC values regardless of the considered instance. In fact, the average of the AUC values is practically equal, as can be seen in the last row of the table. Nevertheless, OpF is almost 5 times faster than WEGA and more than 16 times quicker than OpR.

Finally, similar conclusions than previously can be obtained for the DUD-E database (see Table 4). In terms of effectiveness, OpR and WEGA are comparable, since they obtain practically the same mean AUC value. On the contrary, OpF obtains an average AUC value slightly smaller. Nevertheless, OpF is more than 17 times faster than WEGA and more than 38 times quicker than OpR.

### Results obtained when hydrogen atoms are considered

By default, WEGA does not consider hydrogen atoms during optimization, which is a common practice for most tools in the current scenario, since evaluation without hydrogens is less time-consuming. However, this simplification may have serious consequences in a VS process. In this work, the effect of excluding the hydrogens of the molecules when optimizing is analyzed. Table 5 shows number of atoms for the 40 query molecules selected from the FDA database when the hydrogens are not taken into account and when they are considered (columns 2 and 6 respectively). Additionally, the molecule BestComp from the FDA dataset, which maximizes the shape similarity and the corresponding score value Tc, both when the input molecules include the hydrogens and when they not, is shown. Notice that these experiments were accomplished using OpR since, according to the previous results, it is the most efficient algorithm. For the sake of completeness, the average execution time (in seconds), in both cases, has also been included. As it can be seen, in 15 out of 40 cases, the BestComp molecule differs, depending on whether the hydrogens are considered or not. Additionally, and as expected, the computing time decreases when hydrogens are not considered (see columns 5 and 9). This means that excluding the hydrogens of the molecules is not an appropriate simplification; although the computing effort is shorter, the molecule that which maximizes the shape similarity can change.

**Table 5:** Results obtained by OpR for 40 query compounds from the FDA database.

query	Without hydrogens				With hydrogens				BestComp w/o H evaluated with H
	nA	BestComp	Tc	Time	nA	BestComp	Tc	Time	Tc
DB00529	7	DB00828	0.921	61.2	10	DB09294	0.869	135.5	0.701
DB00331	9	DB01189	0.940	77.4	20	DB09210	0.862	255.8	0.710
DB01352	15	DB00306	0.891	116.5	29	DB00306	0.889	361.1	0.884
DB01365	12	DB00191	0.944	96.7	30	DB00191	0.935	406.9	0.928
DB00380	19	DB00816	0.842	165.1	35	DB01041	0.852	477.4	0.802
DB06216	20	DB00370	0.905	169.2	37	DB00370	0.876	500.1	0.874
DB00693	25	DB01619	0.841	215.2	37	DB01619	0.863	553.4	0.854
DB07615	24	DB01250	0.799	205.4	40	DB00721	0.790	576.5	0.713
DB09219	25	DB00434	0.819	223.1	40	DB01320	0.845	636.2	0.764
DB00674	21	DB01619	0.865	169.9	42	DB01619	0.801	556.7	0.786
DB01198	27	DB00402	0.933	223.9	45	DB00402	0.892	624.7	0.890
DB00887	25	DB06614	0.745	213.5	45	DB00837	0.742	613.0	0.686
DB00246	28	DB01261	0.761	250.7	50	DB01261	0.756	737.6	0.751
DB00381	28	DB01023	0.819	227.5	53	DB01023	0.828	728.6	0.823
DB09237	28	DB01054	0.717	227.4	54	DB01054	0.752	759.4	0.745
DB00876	30	DB09039	0.664	262.0	54	DB09039	0.674	800.8	0.665
DB00254	32	DB00595	0.877	263.1	55	DB00595	0.848	814.7	0.838
DB00351	27	DB04839	0.941	220.7	57	DB04839	0.934	748.3	0.928
DB01196	29	DB00286	0.784	244.7	60	DB00286	0.797	820.4	0.794
DB01621	33	DB01148	0.694	267.1	66	DB01148	0.715	924.2	0.708
DB09236	32	DB00270	0.672	280.7	66	DB01054	0.682	940.2	0.615
DB08903	37	DB00333	0.653	289.3	69	DB00333	0.679	968.5	0.673
DB00632	23	DB00464	0.724	130.4	69	DB00464	0.740	696.4	0.732
DB01419	42	DB06605	0.630	359.2	70	DB06605	0.671	1086.4	0.667
DB00320	43	DB01413	0.629	355.6	80	DB00728	0.617	1139.0	0.596
DB00728	38	DB01339	0.820	284.6	91	DB01339	0.839	1094.0	0.837
DB00503	50	DB00845	0.499	416.6	98	DB00701	0.541	1465.3	0.442
DB01232	49	DB01082	0.549	395.7	100	DB00212	0.617	1411.8	0.581
DB00309	55	DB00541	0.634	377.3	110	DB00541	0.624	1348.2	0.621
DB04786	86	DB01078	0.387	598.7	120	DB00511	0.432	1657.8	0.405
DB09114	50	DB08993	0.476	388.3	130	DB08993	0.512	1799.6	0.510
DB06439	57	DB00207	0.515	434.8	137	DB00207	0.591	1871.5	0.533
DB01078	66	DB00511	0.502	485.9	140	DB00511	0.582	1819.4	0.570
DB01590	68	DB00877	0.469	495.1	151	DB00877	0.557	1995.5	0.545
DB04894	80	DB00364	0.482	550.4	152	DB00646	0.537	1797.1	0.495
DB00403	94	DB00035	0.394	609.5	167	DB08874	0.470	2130.0	0.446
DB00732	87	DB01045	0.434	628.5	169	DB06287	0.484	2204.4	0.470
DB00050	102	DB00569	0.396	664.1	194	DB00569	0.489	2248.0	0.483
DB06699	117	DB00091	0.454	725.6	221	DB09099	0.514	2482.6	0.496
DB06219	128	DB00512	0.422	828.4	229	DB00512	0.443	2796.0	0.414
mean	44		0.686	330.0	86		0.704	1124.6	0.674

Two experiments were carried out, one excluding the hydrogen atoms for all the molecules (a common practice in most VS tools in the literature) and the other hand considering the hydrogens in all the molecules. For each study and query, its nA without and with hydrogens, the BestComp with the highest Tc and the computing time, in second, are shown. Finally, the optimized BestComp obtained when no hydrogens are considered is re-evaluated, but including the hydrogens (last column).

**Figure 10:**
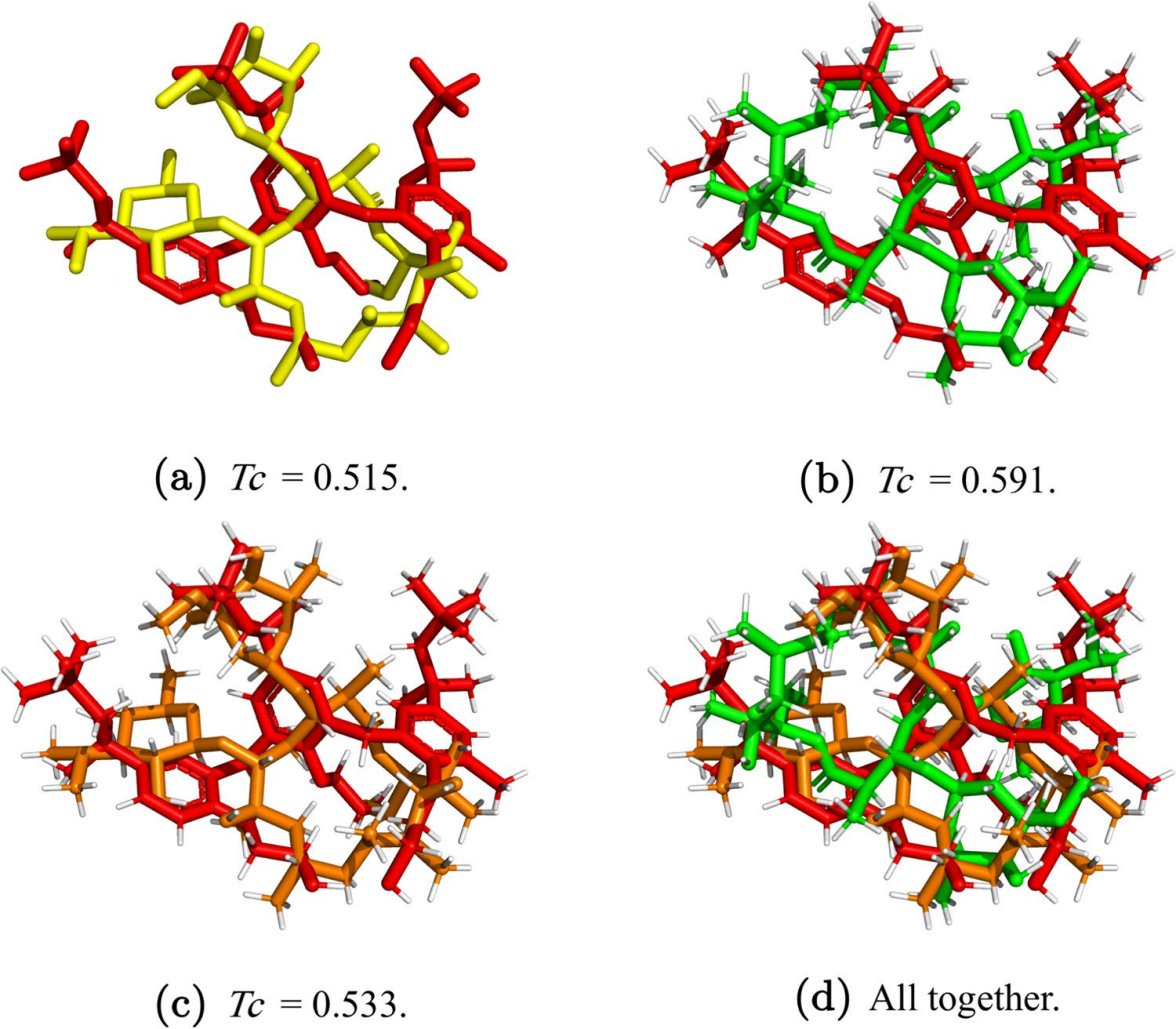
Query compound DB06439 is represented by the red structure. Hydrogens are white atoms. Colours remain fixed. (**a**) *Tc* = 0.515 where the compound DB00207 is the yellow structure. (**b**) *Tc* = 0.591 where the compound DB00207 is the green structure. (**c**) *Tc* = 0.533 where the compound DB00207 is the orange structure. (**d**) The three previous compounds are optimized with respect to the query.

Finally, for a fair comparison in terms of score value, the optimized BestComp obtained by OpR when no hydrogens are considered is re-evaluated, but considering now the hydrogens. As we can see, the obtained score value is always smaller than the one obtained when the hydrogens are included (compare columns 8 and 10). This means that the BestComp molecule found by OpR when the hydrogens are considered is indeed more similar than the one proposed when the hydrogens are excluded. The Fig. 10 illustrates this fact.

In addition, the impact on the classification when the hydrogen atoms are considered has also been evaluated when DUD and DUD-E databases are considered as input. The algorithms OpR and OpF have been selected to this aim. The corresponding results are shown in Tables 6 and 7. Notice that WEGA has not been included in the study since it never considers the hydrogens.

**Table 6:** DUD database with hydrogens.

name	AUC				Time			
	OpR		OpF		OpR		OpF	
	Av	SD	Av	SD	Av	SD	Av	SD
ace	0.40	0.001	0.42	0.021	894.3	8.584	51.3	0.343
ache	0.72	0.002	0.68	0.007	2448.1	95.702	132.8	0.336
ada	0.79	0.006	0.75	0.021	227.9	10.047	15.3	0.092
alr2	0.46	0.007	0.48	0.009	187.4	6.656	15.0	0.073
ampc	0.74	0.013	0.73	0.015	131.4	1.561	9.2	0.021
ar	0.86	0.003	0.84	0.003	748.1	37.040	66.5	0.151
cdk2	0.62	0.003	0.60	0.011	449.6	11.774	30.1	0.143
comt	0.40	0.008	0.41	0.008	136.0	7.498	8.7	0.041
cox1	0.59	0.001	0.58	0.006	141.6	9.842	12.1	0.035
cox2	0.90	0.001	0.88	0.005	3768.5	99.262	237.3	0.578
dhfr	0.59	0.004	0.53	0.007	3946.1	77.654	217.4	0.432
egfr	0.56	0.002	0.57	0.004	5896.4	131.069	379.9	0.484
er_agonist	0.74	0.003	0.71	0.010	751.7	20.644	59.1	0.324
er_antagonist	0.69	0.004	0.73	0.008	887.3	23.421	52.9	0.209
fgfr1	0.42	0.000	0.46	0.002	1782.4	31.747	112.2	0.309
fxa	0.66	0.009	0.61	0.011	3089.2	41.870	166.9	0.443
gart	0.28	0.011	0.34	0.013	469.3	5.374	28.7	0.183
gpb	0.85	0.002	0.82	0.008	329.8	3.580	27.9	0.178
gr	0.77	0.004	0.76	0.011	1222.8	64.358	95.1	0.280
hivpr	0.74	0.010	0.74	0.007	2049.9	94.105	113.9	0.732
hivrt	0.70	0.008	0.69	0.009	470.9	17.540	31.1	0.174
hmga	0.84	0.004	0.82	0.008	855.8	23.483	56.6	0.162
hsp90	0.77	0.012	0.81	0.015	412.6	18.489	26.2	0.063
inha	0.59	0.010	0.53	0.005	1392.1	43.314	89.5	0.289
mr	0.87	0.003	0.86	0.004	235.4	6.255	21.4	0.092
na	0.83	0.002	0.80	0.009	479.8	9.484	40.0	0.275
p38	0.31	0.004	0.37	0.006	6491.8	129.148	346.9	0.598
parp	0.59	0.004	0.59	0.006	232.4	8.260	19.2	0.126
pde5	0.77	0.006	0.75	0.006	1399.2	12.286	78.8	0.473
pdgfrb	0.44	0.004	0.46	0.008	2704.0	93.157	143.2	0.893
pnp	0.71	0.004	0.68	0.017	193.9	1.978	14.9	0.054
ppar_gamma	0.73	0.006	0.73	0.012	3000.9	40.167	139.5	0.172
pr	0.68	0.011	0.66	0.013	544.4	25.760	36.8	0.274
rxr_alpha	0.89	0.023	0.87	0.015	414.1	9.421	25.5	0.152
sahh	0.88	0.006	0.81	0.012	227.1	13.657	15.5	0.036
src	0.44	0.002	0.46	0.005	3727.8	73.533	219.2	0.510
thrombin	0.56	0.010	0.57	0.006	1517.8	16.977	92.9	0.210
tk	0.65	0.003	0.64	0.011	125.6	3.786	11.6	0.065
trypsin	0.27	0.004	0.30	0.008	733.3	10.189	36.3	0.187
vegfr2	0.62	0.003	0.60	0.007	861.6	53.930	54.0	0.280
mean	0.65	0.005	0.64	0.009	1389.5	34.815	83.3	0.262

For each query compound, the average AUC value and the mean running time (in seconds) over 100 independent executions were computed with both OpR and OpF. For the sake of completeness, the SD is also provided for both OpR and OpF versions. The last row of the table shows average values for the query molecules.

**Table 7:** DUD-E database with hydrogens.

name	AUC				Time			
	OpR		OpF		OpR		OpF	
	Av	SD	Av	SD	Av	SD	Av	SD
aa2ar	0.54	0.000	0.54	0.001	12648.0	307.385	256.3	15.165
abl1	0.56	0.000	0.58	0.003	4178.4	107.358	184.4	3.043
ace	0.63	0.000	0.63	0.001	6514.7	169.817	174.3	6.823
aces	0.22	0.000	0.23	0.001	10542.9	234.978	194.1	7.856
ada	0.68	0.000	0.70	0.003	1435.5	50.096	40.2	1.630
ada17	0.53	0.000	0.57	0.001	9711.7	254.865	235.6	1.251
adrb1	0.41	0.001	0.39	0.002	5819.1	180.508	238.1	4.965
adrb2	0.41	0.000	0.42	0.001	8295.1	243.728	167.7	9.623
akt1	0.26	0.001	0.29	0.003	5113.7	105.351	205.4	3.847
akt2	0.47	0.000	0.43	0.002	2762.0	78.460	115.8	2.685
aldr	0.56	0.001	0.55	0.006	2156.1	84.687	87.3	0.626
ampc	0.68	0.000	0.56	0.015	465.2	11.388	12.5	0.532
andr	0.78	0.000	0.75	0.001	3845.4	93.711	196.9	6.801
aofb	0.41	0.000	0.41	0.001	941.4	14.389	24.1	1.065
bace1	0.58	0.000	0.53	0.003	8931.4	248.917	303.5	7.397
braf	0.53	0.000	0.52	0.003	4113.9	105.411	102.8	3.975
cah2	0.50	0.002	0.51	0.001	2636.3	63.463	145.9	4.771
casp3	0.45	0.000	0.48	0.001	2751.9	63.384	88.8	3.199
cdk2	0.64	0.000	0.63	0.002	14337.1	270.933	407.1	8.501
comt	0.63	0.005	0.56	0.003	441.1	12.081	23.5	1.007
cp2c9	0.45	0.000	0.45	0.002	1980.4	33.515	68.4	2.496
cp3a4	0.55	0.000	0.54	0.004	7613.5	271.865	211.5	6.706
csf1r	0.51	0.000	0.54	0.001	5659.4	189.853	146.7	0.106
cxcr4	0.75	0.000	0.69	0.001	1712.4	66.771	30.8	0.612
def	0.76	0.000	0.72	0.002	2013.0	40.488	55.5	1.596
dhi1	0.75	0.000	0.75	0.002	6446.4	158.161	189.5	4.195
dpp4	0.62	0.000	0.61	0.001	15566.7	374.754	328.8	14.946
drd3	0.37	0.000	0.39	0.001	14175.3	269.919	431.7	13.124
dyr	0.42	0.003	0.38	0.002	5729.7	96.321	373.8	4.095
egfr	0.50	0.000	0.51	0.002	18151.4	354.857	336.4	4.806
esr1	0.57	0.001	0.60	0.002	10530.6	293.861	240.5	0.841
esr2	0.64	0.000	0.63	0.003	8166.9	185.100	207.5	5.593
fa10	0.63	0.004	0.61	0.001	13762.4	325.381	628.2	10.031
fa7	0.48	0.001	0.50	0.006	4005.9	117.438	88.6	2.803
fabp4	0.74	0.003	0.67	0.005	1366.2	49.416	51.3	0.834
fak1	0.71	0.001	0.60	0.006	2801.9	81.060	163.6	3.498
fgfr1	0.47	0.002	0.47	0.001	281.7	5.810	9.5	0.613
fkb1a	0.78	0.001	0.73	0.005	2286.2	71.672	72.1	3.259
fnta	0.54	0.001	0.48	0.001	33347.0	569.040	1131.1	13.666
fpps	0.78	0.000	0.75	0.001	902.0	23.601	37.0	2.852
gcr	0.64	0.000	0.62	0.001	5936.8	117.042	194.4	5.002
glcm	0.33	0.003	0.28	0.001	923.6	32.983	21.8	0.044
gria2	0.58	0.000	0.55	0.002	3159.4	95.870	100.3	6.522
grik1	0.54	0.000	0.57	0.004	1198.1	22.338	45.2	4.290
hdac2	0.39	0.000	0.36	0.003	2752.9	88.176	55.1	1.536
hdac8	0.40	0.000	0.36	0.004	3001.7	74.002	83.5	2.987
hivint	0.38	0.000	0.38	0.001	1542.3	51.847	63.8	2.193
hivpr	0.76	0.000	0.73	0.001	26933.4	678.027	764.7	0.995
hivrt	0.56	0.001	0.52	0.001	5961.3	173.759	233.7	9.952
hmdh	0.85	0.000	0.80	0.004	4998.3	136.453	127.5	1.861
hs90a	0.66	0.000	0.65	0.002	1772.9	26.219	56.4	1.329
hxk4	0.65	0.000	0.50	0.003	1488.3	41.273	59.9	2.133
igf1r	0.46	0.001	0.43	0.004	5161.5	144.369	174.9	5.849
inha	0.40	0.000	0.42	0.004	680.8	28.818	11.7	0.104
ital	0.41	0.002	0.44	0.003	5063.5	158.899	129.6	0.267
jak2	0.72	0.000	0.69	0.002	2058.1	58.984	48.4	3.409
kif11	0.83	0.000	0.68	0.003	3439.9	123.658	54.4	3.115
kit	0.38	0.000	0.38	0.001	3238.8	110.014	91.8	8.589
kith	0.72	0.001	0.69	0.003	766.2	9.368	24.1	0.997
kpcb	0.57	0.000	0.53	0.005	3258.8	90.824	94.7	2.935
lck	0.41	0.000	0.40	0.001	11895.8	307.804	247.0	12.443
lkha4	0.57	0.000	0.57	0.003	3373.8	105.163	51.1	0.528
mapk2	0.63	0.001	0.61	0.001	1820.0	67.425	60.5	3.590
mcr	0.78	0.000	0.73	0.001	1616.9	48.168	46.3	2.394
met	0.71	0.005	0.68	0.005	11546.8	450.466	250.3	6.843
mk01	0.44	0.000	0.39	0.002	1259.4	33.755	39.0	1.265
mk10	0.45	0.000	0.46	0.002	2520.8	86.614	70.2	2.139
mk14	0.54	0.001	0.53	0.002	28472.3	565.522	692.5	14.399
mmp13	0.61	0.000	0.58	0.000	18288.5	482.792	358.3	18.123
mp2k1	0.45	0.000	0.54	0.001	3429.1	71.075	69.3	6.095
nos1	0.34	0.000	0.35	0.001	2571.5	73.863	58.7	2.016
nram	0.88	0.000	0.86	0.002	1839.6	46.528	43.6	3.260
pa2ga	0.67	0.000	0.66	0.004	2310.8	67.884	59.1	5.073
parp1	0.64	0.000	0.66	0.000	7534.6	164.474	261.3	7.200
pde5a	0.50	0.000	0.48	0.003	7966.8	132.170	127.7	0.662
pgh1	0.70	0.000	0.70	0.003	3045.1	89.026	102.0	5.014
pgh2	0.72	0.000	0.70	0.002	4841.0	142.901	201.6	7.323
plk1	0.60	0.000	0.54	0.003	4137.3	131.707	59.5	0.316
pnph	0.72	0.000	0.67	0.005	1321.8	49.860	29.9	0.095
ppara	0.67	0.000	0.65	0.002	9214.1	279.082	184.1	2.299
ppard	0.39	0.001	0.37	0.006	7555.7	234.371	194.5	4.637
pparg	0.41	0.000	0.37	0.001	13606.9	308.089	388.7	12.057
prgr	0.80	0.000	0.75	0.004	5894.0	155.765	208.6	4.872
ptn1	0.35	0.000	0.36	0.002	1233.6	29.355	36.5	2.168
pur2	0.47	0.000	0.37	0.007	1035.2	31.177	28.7	1.284
pygm	0.61	0.000	0.62	0.002	1091.7	30.718	36.0	1.144
pyrd	0.81	0.000	0.81	0.004	1474.6	37.570	34.4	0.277
reni	0.68	0.001	0.65	0.005	6085.1	234.368	253.1	3.662
rock1	0.56	0.000	0.56	0.001	1414.9	44.631	26.4	0.190
rxra	0.72	0.000	0.55	0.006	2236.7	58.555	50.5	0.268
sahh	0.85	0.000	0.61	0.002	549.8	12.608	9.6	0.279
src	0.57	0.003	0.55	0.001	23435.6	686.152	1187.2	15.975
tgfr1	0.53	0.000	0.53	0.001	2329.2	76.982	48.8	0.365
thb	0.79	0.000	0.75	0.003	3150.5	82.192	83.2	3.025
thrb	0.50	0.000	0.48	0.002	13973.6	228.488	444.4	3.482
try1	0.59	0.000	0.60	0.002	12992.8	261.488	384.5	0.510
tryb1	0.42	0.000	0.38	0.004	3221.7	92.226	63.8	2.376
tysy	0.60	0.000	0.58	0.004	3038.2	113.099	72.7	0.099
urok	0.38	0.000	0.37	0.001	2944.9	92.744	77.0	0.357
vgfr2	0.52	0.000	0.54	0.001	9023.2	150.967	242.1	5.486
wee1	0.70	0.001	0.56	0.002	3547.3	120.772	77.2	2.831
xiap	0.86	0.000	0.80	0.005	3272.1	112.115	101.8	4.684
mean	0.58	0.000	0.55	0.003	5878.3	148.367	171.6	4.183

For each query compound, the average AUC value and the mean running time (in seconds) over 100 independent executions were computed with both OpR and OpF. For the sake of completeness, the standard deviation SD is also provided for both OpR and OpF versions. The last row of the table shows average values for the query molecules.

Broadly speaking, the mean AUC value increases slightly when the hydrogen atoms are considered in DUD database, for both OpR and OpF algorithms. See last row of Tables 3 and 6. In particular, an increment of 0.03 (resp. 0.01) has been obtained for OpR (resp. OpF). In addition, for 23 out of 40 cases, OpR obtains better AUC values when the hydrogens are considered. Regarding OpF, it happens for 20 out of 40 instances.

The same increasing tendency can be appreciated in the mean AUC value when the DUD-E database is considered. Please, see Tables 4 and 7. In this case, an increment of 0.02 has been obtained for both OpR and OpF algorithms. Both OpR and OpF obtain better AUC values in more than half of the cases (58 out of 102 for OpR and 67 out of 102 for OpF).

In general terms, considering the hydrogens increases the average computing time. Compare again Tables 3 and 6 for DUD database, and Tables 4 and 7 for DUD-E benchmark. As can be seen, the time increases 2.9x times for both OpR and OpF when DUD is considered as input. For the DUD-E case, the increase is of 4.8x and 5.7x for OpR and OpF, respectively. Of course, the larger the number of atoms considered for a compound, the higher the computing time associated to its evaluation, but the more realistic the associated scoring function value.

Therefore, based on the results, it can be concluded that a more realistic classification of compounds can be obtained if hydrogen atoms are considered. In such a case, the computing time can be reduced by using high-performance computing approaches.

### Results obtained for Maybridge database

Finally, a study has been conducted to show the utility of OpR, i.e. it can find good quality solutions when possible.

The effectiveness of OpR has been analyzed when it is executed with the Maybridge database considering hydrogens. In particular, a set of query compounds were selected from such a database. The choice procedure was carried out as follows: the Maybridge dataset was initially sorted according to the number of atoms of the compounds and split into 38 intervals. Then, a single compound was randomly chosen for each interval. Table 8 summarizes the obtained results. In particular, it is shown: (i) the number nC of compounds with a number of atoms included in the interval ${\rm{nA}}\in [i,j)$; (ii) the randomly selected query from such an interval, and (iii) the other molecule from Maybridge (BestComp) with the highest shape similarity value (Tc) according to OpR. The last row of the table shows the total number of compounds with nA < 95 (resp. nA ≥ 95) and the average Tc value. Notice that there exist intervals with 0 compounds, we note those cases by including ‘−’ in the corresponding columns.

**Table 8:** Maybridge database.

[i, j)	nC	Queries with nA < 95			[i, j)	nC	Queries with nA ≥ 95		
		query	BestComp	Tc			query	BestComp	Tc
[0, 5)	0	—	—	—	[95, 100)	6	JFD01206	JFD01203	0.930
[5, 10)	2	CD08226	RF01682	0.940	[100, 105)	3	JFD00633	JFD01915	0.875
[10, 15)	93	AC10702	KM03331	0.982	[105, 110)	3	JFD02451	JFD02452	0.762
[15, 20)	968	AC10402	RF03315	0.939	[110, 115)	3	JFD01915	JFD00633	0.877
[20, 25)	3469	AC11546	NRB00891	0.940	[115, 120)	1	JFD02945	RH00477	0.512
[25, 30)	7050	AC10751	AC11968	0.991	[120, 125)	2	BTB14731	JFD01602	0.508
[30, 35)	10414	AC12586	RH01548	0.895	[125, 130)	1	JFD01714	JFD01716	0.676
[35, 40)	10623	AC10018	JFD00624	0.939	[130, 135)	0	—	—	—
[40, 45)	9015	AC10608	HTS01369	0.867	[135, 140)	1	JFD02946	RJC01701	0.474
[45, 50)	6085	AW00180	AW00174	0.873	[140, 145)	0	—	—	—
[50, 55)	3008	AW00136	HTS03294	0.849	[145, 150)	1	JFD02949	JFD00655	0.552
[55, 60)	1479	JFD00968	RJC02093	0.993	[150, 155)	2	BTB12204	BTB12205	0.600
[60, 65)	648	JFD03035	NRB03291	0.972	[155, 160)	2	BTB12205	BTB12204	0.600
[65, 70)	247	HTS13346	HTS13343	0.982	[160, 165)	1	RJC01719	BTB12214	0.487
[70, 75)	108	JFD01818	RJC03231	0.976	[165, 170)	2	RJC01701	JFD02451	0.645
[75, 80)	57	JFD01718	JFD01716	0.957	[170, 175)	0	—	—	—
[80, 85)	50	NRB03718	NRB03775	0.991	[175, 180)	0	—	—	—
[85, 90)	40	JFD00292	JFD00294	0.877	[180, 185)	1	JFD02950	JFD00655	0.417
[90, 95)	14	JFD01716	JFD01718	0.959	[185, 190)	0	—	—	—
mean	53370			0.940		29			0.637

The number nC of queries from the database with a number of atoms ${\rm{nA}}\in [i,j)$ is shown. From each interval, a query has been randomly selected, and the other molecule from the database (BestComp) with the highest Tc has been computed by using OpR. Note that the score Tc is equal to 1 when the query compound is compared with itself for all the instances, so that BestComp really represents the second most similar molecule to the query.

As can be seen in Table 8, OpR obtains an average Tc value of 0.940 for queries with nA < 95. This is not rare since the number of compounds with less than 95 atoms is equal to 53370, so the probability of finding similar molecules is relatively high. On the contrary, the average Tc value obtained by OpR for molecules with more than 95 atoms is equal to 0.637, which is not a bad figure if we consider that only 29 out of 53399 molecules have more than 95 atoms. Even so, OpR obtains good quality solutions for queries with more than 95 atoms. See for example, the instances JFD0120 and JFD0063, with 96 and 104 atoms, respectively. For those two cases, OpR has found compounds with Tc values of 0.930 and 0.875, even when the number of molecules with similar sizes is not high. Let focus now on the worst cases, i.e. those where OpR obtains the lowest Tc values. They are JFD02950 and JFD02946 with 180 and 135 atoms, respectively. Notice that there are not molecules in the database with similar sizes. More precisely, there are just 10 molecules, including JFD02950 and JFD02946, with ${\rm{nA}}\in [135,190)$. Therefore, the probability of discovering similar molecules in terms of shape is very low, since the most likely is that they do not exist. Then, from the results, it is possible to infer that OpR finds a high-quality solution to a given query when it exists in the corresponding database.

## Conclusions and Future Work

This work has introduced the SSM OptiPharm, based on novel metaheuristic approaches and illustrated its performance in terms of prediction accuracy and running time when processing well-known benchmarks such as DUD, and in addition FDA dataset. Comparison made with WEGA show that OptiPharm offers the same predictive accuracy but at a much lower computational cost (average speedup is 5x). Another of the advantages of the method compared with WEGA is that its optimization algorithm is easily parameterizable so that very different heuristic schemes can be tested, and so it adapts itself to a given database depending on the average molecular size and topology, to name a few. Also, bearing in mind that OptiPharm, unlike WEGA, allows optimizing including the hydrogen atoms of the compounds. Results have shown that its consideration improves the predictions, although it is more costly from a computational point of view. High-performance computing approaches may be a good alternative to deal with this drawback.

OptiPharm has been designed with parallelism in mind. Notice that each pose in the population can generate a new offspring without the participation of the remaining quaternions in the population, meaning that the Reproduction method can be easily parallelized by dividing the poses in the population among the available processing units. Similarly, the poses can also be enhanced by distributing them in the Improvement procedure. This means that OptiPharm can be drastically accelerated by using high-performance computing with practically no effort. In the future, several programming paradigms based on both shared and distributed memory architectures will be implemented and analyzed. In particular, a parallel version of OptiPharm will be implemented to be executed on GPUs, and compared with the GPU-accelerated WEGA^58^.

## Data Availability

The databases belong to their authors and access to them depends on any applicable restrictions. OptiPharm software is available upon request via email.
